# Embodied cognition-driven interpretable trajectory prediction of autonomous systems

**DOI:** 10.1038/s41467-026-75091-9

**Published:** 2026-07-06

**Authors:** Xiao Wang, Quancheng Du, Qiong Wu, Xiaofeng Jia, Liang Lin, Ljubo Vlacic, Changyin Sun, Fei-Yue Wang

**Affiliations:** 1 School of Robotics(Institute of Embodied Intelligence), Anhui University, Hefei, Anhui China; 2 Engineering Center of the Ministry of Education for Autonomous Unmanned Systems Technology, Anhui University, Hefei, Anhui China; 3 School of Computer and Information Engineering, Henan Normal University, Xinxiang, Henan China; 4 Anhui Jianghuai Automobile Group Corp. Ltd, Hefei, Anhui China; 5 Beijing Big Data Center, Beijing, China; 6 School of Computer Science and Engineering, Sun Yat-sen University, Guangzhou, China; 7 Institute of Intelligent and Integrated Systems, Griffith University, Brisbane, QLD Australia; 8 Anhui Province Engineering Center of Unmanned Systems and Intelligent Technology, Anhui University, Hefei, Anhui China; 9 Faculty of Innovation Engineering, Macau University of Science and Technology, Macau, China; 10 DeSci Center of Parallel Intelligence, Obuda University, Budapest, Hungary

**Keywords:** Decision making, Electrical and electronic engineering, Civil engineering

## Abstract

For autonomous systems to operate safely and reliably in dense traffic, they must perform trajectory prediction with human-like, interpretable reasoning. Prevailing data-driven “black-box” models fundamentally lack this capability. This research proposes a paradigm shift toward embodied intelligence, unifying cognitive science principles into a hierarchical framework: a Scene Attention Mechanism for threat prioritization, Social Impact Theory-driven graphs for intent inference, and a physics-compliant Social Force Model. Experimental results demonstrate that our framework reduces average displacement error by 42% and Final Displacement Error by 40% compared to existing state-of-the-art models on ETH and UCY, while enabling near-real-time inference (0.003 s). Crucially, the model’s interpretable architecture, which is validated through risk-sensitive heatmaps and graph visualizations, reveals how agents dynamically balance safety, efficiency, and socio-cultural norms. Beyond performance gains, this work constructs an interpretable bridge between computational models and human cognitive science, laying a foundation for trustworthy autonomous systems.

## Abstract (web summary)

The study presents an interpretable method for predicting future paths of vehicles and pedestrians by combining scene-level attention, graph-based social interaction reasoning, and physics-based motion constraints, improving accuracy, safety, and speed while enabling transparent decisions.

## Introduction

**Fig. 1:**
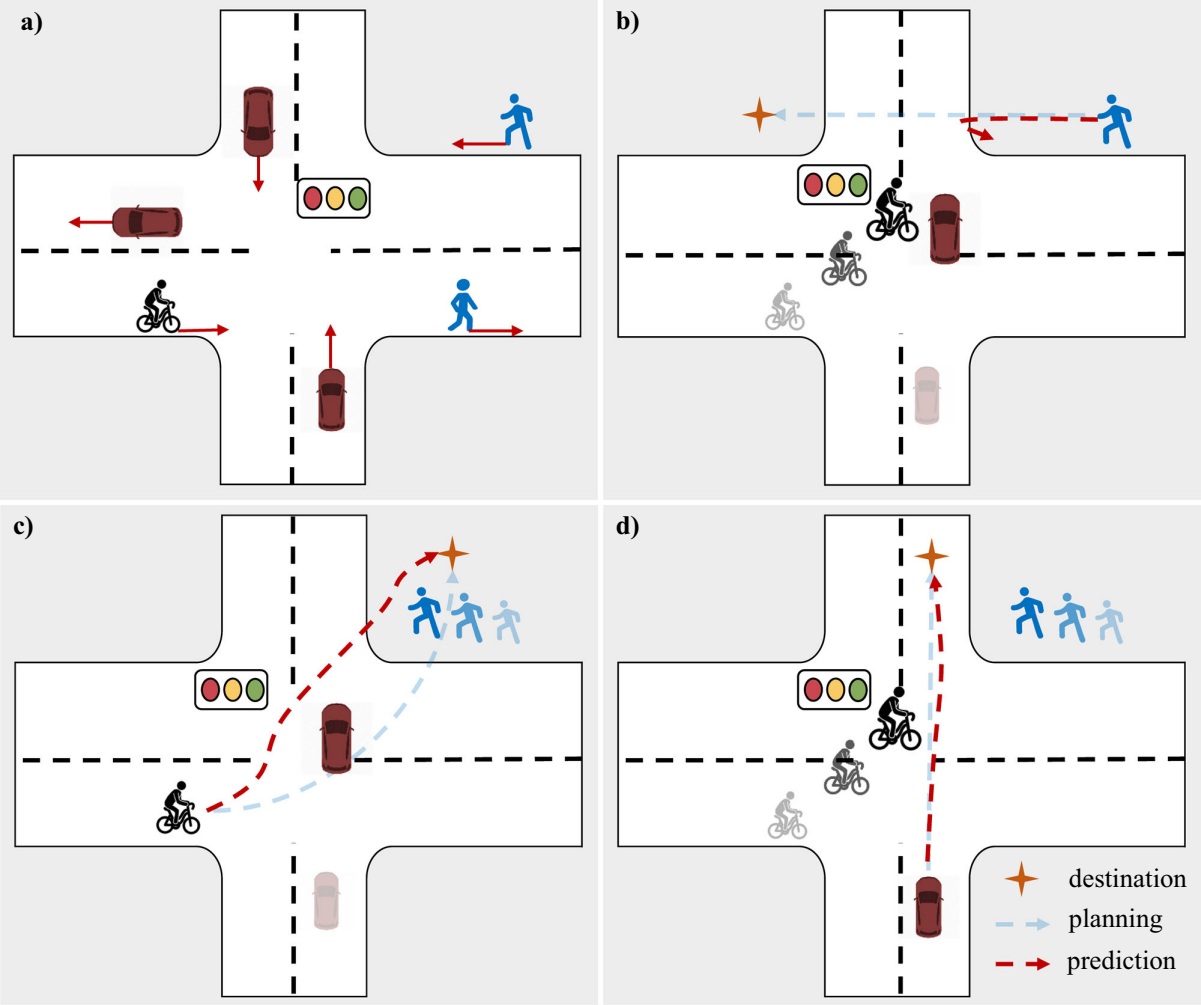
The non-smooth trajectories of different traffic participants at an intersection. **a** The overhead view of movement patterns. **b** Pedestrians' non-smooth walking path due to the approaching vehicles. **c** Bicycle’s swerving path to avoid a car. **d** Car’s adjusting speed and direction in response to nearby agents.

The human situation cognition process in dense and dynamic traffic generally includes the perception of others’ positions, speeds, and directions (e.g., the steering angle of a vehicle, the gait of a pedestrian), using environmental context to infer likely actions and conflicts^1,2^. Subsequently, it involves predicting how the traffic situation will evolve and making proactive responses to avoid collisions while ensuring safety and efficiency. While situated cognition emphasizes how external context(social, cultural, environmental) shapes thought, embodied cognition focuses on internal bodily processes (sensorimotor experiences, physiology)^3–5^. For autonomous systems, accurate prediction of agents’ future trajectories is paramount because it enables real-time adaptation to uncertainty, ensures collision-free navigation, and aligns with the human cognitive principle of safety-first decision-making. Although data-driven models (e.g., LSTMs, Transformers) excel at pattern recognition, they neglect the WHY behind actions-specifically, how sensory inputs, social dynamics, and biomechanics interact. This limits their reliability in culturally diverse, safety-critical scenarios^6,7^. For humans navigating complex and dynamic traffic environments filled with diverse participants, success hinges not only on perceiving physical obstacles but also on interpreting and anticipating the behavior of others, which is inherently nuanced and uncertain^8,9^. Figure 1 illustrates the time-varying trajectories of pedestrians, bicycles, and cars at an intersection from different views, reflecting the complexity of interactions among various traffic participants.

Human trajectory prediction methods can be categorized into model-driven and data-driven approaches^10,11^. Early research predominantly utilized model-driven methods to simulate pedestrian movement patterns, encompassing kinematic models^12^, dynamic models^13^, Gaussian processes^14^, Markov decision process^15^, and Bayesian networks^16^. However, the performance of these methods is constrained by their dependency on manually designed parameters and their inability to fully account for social interactions between pedestrians, leading to insufficient accuracy in complex scenarios. In recent years, the rapid development of deep learning has enabled data-driven methods to demonstrate significant potential^17,18^. These methods can extract pedestrian motion features from vast amounts of real-world data, such as videos, to predict trajectories with greater precision. For example, Alahi et al.^19^ proposed the Social-LSTM model, a representative application of recurrent neural networks (RNNs) in pedestrian trajectory prediction (PTP). It integrates the hidden states of neighbors through grid pooling to simulate social interactions among pedestrians. Gupta et al. ^20^ introduced generative adversarial networks (GANs) on this foundation, constructing a pooling mechanism in the Social-GAN model to determine interactive relationships. Despite these advancements, RNN-based models still face challenges such as vanishing or exploding gradients when processing long-sequence data. To more accurately capture the interaction differences among various agents, Sadeghian et al. ^21^ and Kosaraju et al. ^22^ introduced attention mechanisms for interaction modeling, assigning differentiated importance weights to multi-class agents to obtain refined interaction information. Although attention mechanisms enable models to recognize behavioral diversity, they do not fully consider the information transmission process among pedestrians^23^. Recent works^24,25^ have employed graph-based methods, which adaptively aggregate neighbor features through adjacency matrices to learn social interactions. These methods provide an effective means to represent the topology of pedestrians in shared spaces and better simulate social interactions within a scene. STGAT^24^ leverages a graph attention network (GAT) to learn interaction weights between pedestrians and applies GAT directly to the hidden states of a long short-term memory (LSTM) network to capture spatial interactions. Social-STGCNN^25^ embeds the social interaction behaviors of pedestrians into an adjacency matrix, models it as a graph network structure, and processes the matrix with a kernel function to capture spatio-temporal interactions.

Based on the reviewed literature, the primary limitations of current trajectory prediction methods can be summarized as follows:

- Lack of interpretability: Predominantly data-driven models (e.g., LSTMs, transformers) excel at pattern recognition but operate as black boxes. They neglect the WHY behind actions-failing to explain how sensory inputs, social dynamics, and biomechanics interact to influence decisions. This limits their reliability and trustworthiness in safety-critical scenarios^6,7,26^.
- Inadequate socio-cognitive modeling: While social force models (SFM)^13^ incorporate physical constraints, they lack dynamic, intent-aware social reasoning. Recent graph-based approaches^27^ have advanced interaction modeling but do not explicitly embody human cognitive principles such as attentional filtering^28^ or the nuanced balance of social impact factors (strength, immediacy, number), which can be formalized by social impact theory (SIT)^29^.
- Inflexibility to social and cultural context: Existing approaches often lack mechanisms to adapt to culturally influenced social norms (e.g., varying pedestrian priority rules across regions). This inflexibility limits their reliability and safety in diverse, real-world ecosystems ^6,30^.
- Computational inefficiency vs. safety trade-off: Achieving high accuracy often comes at the cost of computational complexity, hindering real-time application ^31^. Conversely, simpler models may fail to capture critical, high-risk interactions, especially those involving occluded agents or complex multi-class scenarios, leading to potential safety oversights^32^.

These limitations collectively underscore the necessity for a new paradigm. This paradigm must move beyond purely data-driven pattern matching toward an interpretable, cognitively-inspired framework capable of human-like reasoning and adaptability.

Embodied cognition posits that cognitive processes, such as scene perception, reasoning and prediction, emerge from dynamic interactions between sensory perception, biomechanical constraints, and environmental affordances^3,33^. In dense and diverse traffic scenarios, human trajectory prediction epitomizes this interplay. Here, agents continuously adapt their movements to balance collision risks, social norms, and physical laws ^34–36^. Although social norms and traffic laws exhibit sociogeographic variability, the neurocognitive capacity to dynamically balance these factors—weighing trust in others’ intentions against biomechanical constraints—represents a universal feature of human cognition in complex environments^37,38^. Critically, human trajectories emerge not as deterministic responses to observed stimuli, but as adaptive deviations shaped by iterative prediction-error minimization. This reflects the embodied nature of human decision-making under uncertainty^39,40^.

**Fig. 2:**
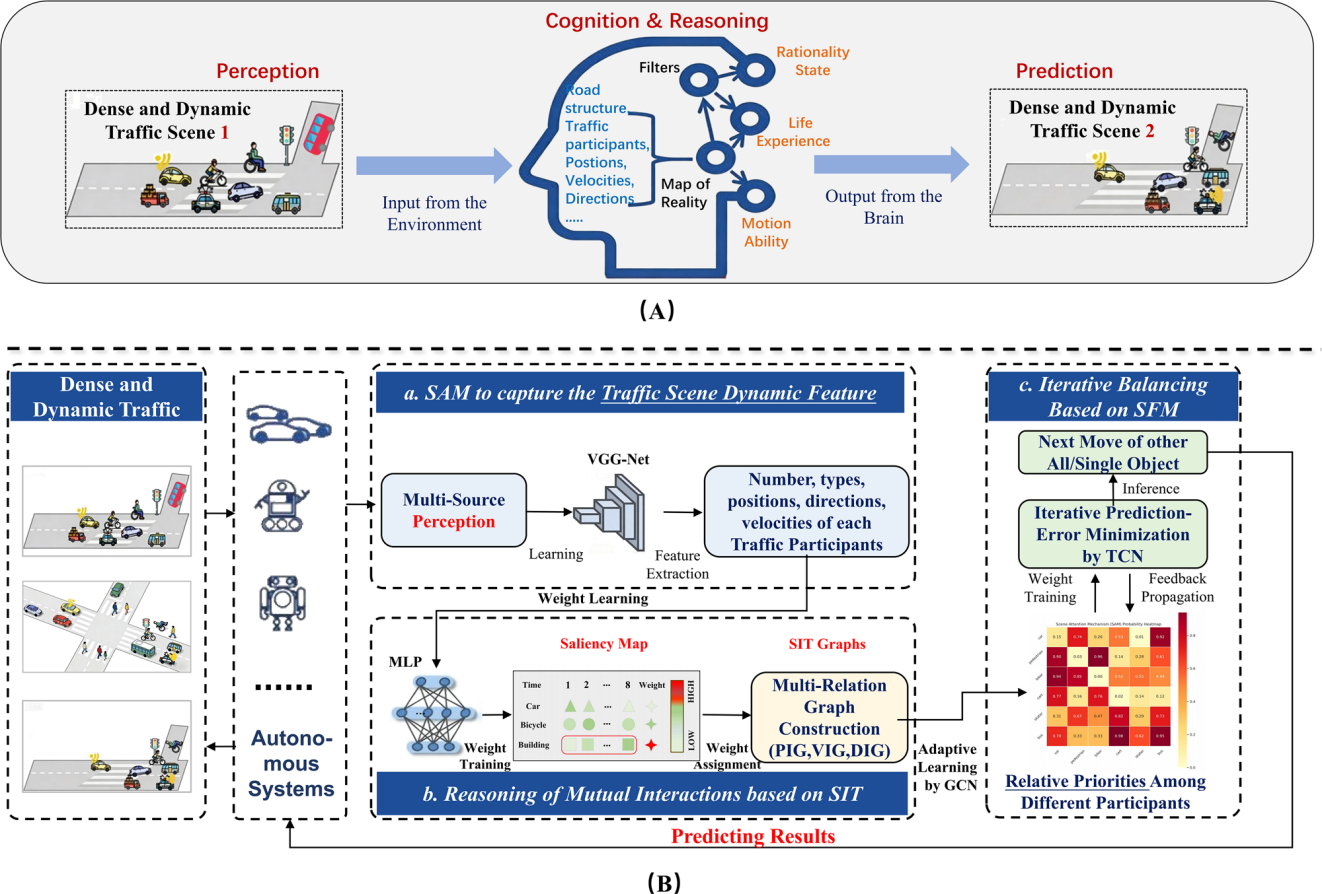
Comparison of human and autonomous system cognition processes for traffic scenarios. **A** Human perception-to-prediction process. **B** Autonomous system’s intelligent perception-to-prediction process inspired by human cognition.

To construct a human-like embodied cognition process, we synergize three components: a scene attention mechanism (SAM) that mimics human visual perception, an SFM that simulates Newtonian force interactions, and SIT to model how humans are impacted by and balance multiple considerations in dynamic, open environments. We integrate these into an embodied cognition paradigm for human trajectory prediction, as illustrated in Fig. 2. Specifically, this paradigm operates through: (1) SAM for Sensory Filtering: SAM dynamically prioritizes high-risk agents (e.g., fast-moving vehicles) while filtering irrelevant stimuli, which mimics human peripheral vigilance^28,41^. (2) SIT for Social Cognition Embodiment: SIT models how proximity, velocity, and intent conflicts shape collective behavior. We encode Latane’s^29^ SIT into graph networks. (3) SFM for Biomechanical Interactions: SFM modulates trajectories to respect physical constraints (e.g., inertia, collision avoidance)^13^, by formalizing social impact into Newtonian forces.

These components mirror findings in behavioral psychology, where humans dynamically allocate attention to proximate and high-risk agents, akin to peripheral vigilance in crowded environments^42^. For instance, studies show that pedestrians prioritize agents within a 2–4 m radius-a phenomenon replicated in our position impact graph (PIG) through inverse-distance weighting^28^.

This is the paradigm that unifies sensory filtering, social cognition, and biomechanical realism under an embodied cognition paradigm. The unification enables human-like adaptability in dense/diverse traffic, where agents balance competing cues (e.g., goal-directed motion vs. collision avoidance) through iterative prediction-error minimization, a process mirroring human cognitive processes ^34^. As visualized in Fig. 2, our paradigm addresses a critical gap in trajectory prediction. Prevailing models often prioritize isolated dimensions (e.g., kinematic accuracy at the expense of sociobehavioral intentionality), which limits their generalizability and contextual alignment^26^. By synthesizing these axes, our paradigm advances the field through mechanistically interpretable modeling, bridging theoretical insights from social psychology and cognitive science with computational innovation^43^. This not only enhances predictive accuracy but also fosters the development of autonomous systems capable of context-aware, ethically grounded decision-making, which is an essential step toward equitable human-AI collaboration in safety-critical environments^44,45^. To further illustrate the unified embodied cognition paradigm for autonomous systems, we elaborate the key aspects.

To address the limitations, this paper proposes an embodied cognition-driven framework. Our main contributions are:

1. Bridging the interpretability gap: We synergize a SAM for human-like sensory filtering, SIT for intent-aware social reasoning via multi-relational graphs, and a physics-compliant SFM for biomechanical realism. This unified, white-box architecture provides mechanistic explanations for predictions through risk-sensitive heatmaps and graph visualizations.
2. A proposed computational model of social dynamics: this is the work to formalize SIT into a data-driven, quantifiable computational model (via PIG, VIG, DIG graphs) for trajectory prediction, enabling explicit modeling of strength, immediacy, and number of social influences.
3. Closing the perception-cognition-action loop: We introduce the unified architecture that integrates perception, cognition, and action within an iterative prediction-error minimization framework. It integrates sensory filtering (SAM), socio-cognitive reasoning (SIT), and physics-based action (SFM) into a single iterative prediction-error minimization process, mirroring human cognitive adaptation in dense traffic.
4. State-of-the-art performance with efficiency: Our framework achieves superior accuracy (42% and 40% reductions in ADE and FDE) on benchmarks (ETH, UCY, SDD, PIE, JAAD) while enabling near-real-time inference (0.003 s), demonstrating a superior trade-off between safety and computational efficiency.

## Results

To validate our proposed framework-which integrates the SAM for threat prioritization, SIT via multi-relational graphs for intent reasoning, and a modulated SFM for physical realism-we conducted extensive experiments addressing three key questions: predictive accuracy, interpretability, and generalizability. Detailed methodologies are provided in Section “Methods”. Below, we introduce the datasets and metrics (Sections “Datasets” and “Evaluation metrics”, followed by quantitative and qualitative analyses.

### Datasets

To comprehensively evaluate the performance and generalizability of the proposed framework, we conducted experiments on several widely adopted benchmark datasets. These can be categorized into two groups based on their data collection perspective: overhead-view datasets (ETH^46^/UCY^47^ and SDD^48^) and ego-view datasets (PIE^49^ and JAAD^50^). The characteristics of each dataset are described below.

ETH/UCY datasets: The ETH (also known as the ETH Walking Pedestrians datase) and UCY are widely adopted benchmarks for evaluating trajectory prediction models in dense, dynamic environments. The ETH dataset comprises two distinct scenarios: ETH, which is captured in a university campus plaza scenario; and HOTEL, which is captured in a hotel lobby scenario. Both of them capturing pedestrian interactions in structured, high-density settings. The UCY dataset extends this diversity with three scenarios: ZARA1 and ZARA2, which collect data on outdoor pedestrian zones near retail stores; and UNIV, which is collected at a university courtyard with complex crowd flows. Collected via overhead cameras, both datasets provide precise, real-world trajectories of pedestrians navigating socially nuanced scenarios, including collision avoidance, group movement, and abrupt directional changes.

Stanford drone dataset (SDD): To further validate the generalization ability of the proposed framework, we conducted additional experiments on SDD. SDD is a benchmark resource for studying multi-agent trajectory prediction in complex, real-world traffic ecosystems. Captured via aerial drones over Stanford University’s campus, the dataset provides high-resolution overhead views of dense, dynamic interactions among six distinct agent categories: pedestrians, cyclists, cars, carts, buses, and skaters. Scenarios include unstructured intersections, shared zones, and pedestrian plazas, where agents navigate with heterogeneous kinematics and socially contingent behaviors (e.g., cyclists swerving around pedestrians, cars yielding at unmarked crosswalks). Trajectories are segmented into 8-second clips. The SDD’s overhead perspective introduces challenges such as occlusion reasoning, velocity estimation for small agents (e.g., bikes), and directional ambiguity in crowded scenes. Its diversity in agent types and interaction patterns makes it indispensable for evaluating models’ capacity to generalize across culturally influenced, safety-critical scenarios.

PIE and JAAD datasets: The pedestrian intention estimation (PIE) dataset and the joint attention for autonomous driving (JAAD) dataset provide ego-centric video data captured from the perspective of a moving vehicle. They offer rich annotations for pedestrian behavior, intention, and trajectory prediction in urban driving scenarios. Both datasets include detailed labels such as pedestrian bounding boxes, intention states (e.g., crossing/not crossing), gaze direction, and contextual cues (e.g., traffic lights, crosswalks). These enable the evaluation of trajectory prediction models in realistic, safety-critical autonomous driving settings where perception is partial and interactions are viewed from the vehicle’s viewpoint. In this work, following previous studies^51^, we discarded trajectories shorter than 2 seconds and used 0.5 s of observation data, as input to predict future trajectories of lengths 0.5, 1.0, and 1.5 s.

### Evaluation metrics

To comprehensively evaluate the performance of trajectory prediction models, we employ several widely adopted metrics. These metrics measure the discrepancy between predicted trajectories and ground truth, with lower values indicating better performance.

Average displacement error (ADE) and final displacement error (FDE): consistent with other baselines, we evaluated the model using the ADE and FDE metrics introduced in refs. ^19,25^, which is widely used in the field. ADE measures the average prediction error across all time steps, while FDE focuses on the error at the final time step. ADE and FDE are used as standard metrics which are defined as:

1 $$ADE=\frac{{\sum }_{i=1}^{N}\mathop{\sum }_{t={T}_{{{\rm{obs}}}}+1}^{{T}_{{{\rm{obs}}}}+{T}_{{{\rm{pred}}}}}{\left\Vert {Y}_{t}^{i}-{\widehat{Y}}_{t}^{i}\right\Vert }_{2}}{\,N\times {T}_{{{\rm{pred}}}}}$$

2 $$FDE=\frac{\mathop{\sum }_{i=1}^{N}{\left\Vert {Y}_{t}^{i}-{\widehat{Y}}_{t}^{i}\right\Vert }_{2}}{\,N},t={T}_{{{\rm{obs}}}}+{T}_{{{\rm{pred}}}}$$

where *N* is the number of agents, ${Y}_{t}^{i}$ represents the ground truth of the future trajectories, and ${\hat{Y}}_{t}^{i}$ denotes the predicted trajectory of the *i*-*th* agent.

CADE and CFDE: For datasets that provide bounding box annotations (e.g., PIE and JAAD), we also report the CADE and CFDE. These metrics measure the error specifically at the center point of the bounding box, offering a more precise evaluation for object detection-oriented applications. Given the ground truth bounding box center ${y}_{t}^{i}$ and the predicted center ${\hat{y}}_{t}^{i}$ for agent *i* at time *t*, CADE and CFDE are defined as:

3 $$CADE=\frac{\mathop{\sum }_{i=1}^{N}\mathop{\sum }_{t={T}_{{{\rm{obs}}}}+1}^{{T}_{{{\rm{obs}}}}+{T}_{{{\rm{pred}}}}}{\left\Vert {y}_{t}^{i}-{\hat{y}}_{t}^{i}\right\Vert }_{2}}{N\times {T}_{{{\rm{pred}}}}}$$

4 $$CFDE=\frac{\mathop{\sum }_{i=1}^{N}{\left\Vert {y}_{{T}_{{{\rm{obs}}}}+{T}_{{{\rm{pred}}}}}^{i}-{\hat{y}}_{{T}_{{{\rm{obs}}}}+{T}_{{{\rm{pred}}}}}^{i}\right\Vert }_{2}}{N}$$

These metrics reflect the model’s ability to accurately predict the central location of agents, which is crucial for safety-critical applications like autonomous driving, where precise localization is essential.

Near-collisions rate (NCR): To evaluate the model’s ability to generate safe and socially compliant trajectories that respect personal space, we introduce the NCR as an open-loop collision-aware safety metric. It measures the probability of predicted trajectories resulting in two agents coming within a critical distance threshold *d*_crit_ over the prediction horizon. Following common practices in pedestrian interaction studies, we set *d*_crit_ = 0.1 m. For a set of *N* agents and *T*_pred_ prediction steps, NCR is calculated as:

5 $${{\rm{NCR}}}=\frac{1}{N\cdot {T}_{{{\rm{pred}}}}}\mathop{\sum }_{t={T}_{{{\rm{obs}}}}+1}^{{T}_{{{\rm{obs}}}}+{T}_{{{\rm{pred}}}}}{\sum }_{i=1}^{N}{\sum }_{j\ne i}^{N}{\mathbb{I}}(\parallel {{{\bf{p}}}}_{t}^{i}-{{{\bf{p}}}}_{t}^{j}{\parallel }_{2} < {d}_{{{\rm{crit}}}})$$

where ${\mathbb{I}}(\cdot )$ is the indicator function, and ${{{\bf{p}}}}_{t}^{i}$ is the predicted position of agent *i* at time *t*. A lower NCR indicates better collision avoidance capability and a higher degree of social realism in the predicted paths.

### Implementation details

We implemented the proposed model using the PyTorch deep learning framework^52^. The NVIDIA GeForce RTX 4090 GPU was used for training and evaluation. Stochastic Gradient Descent (SGD) was employed as the neural network optimizer. The initial learning rate was set to 0.01, and the decay was set to 0.002 after 150 epochs. The PReLU activation function was utilized in the model.

### Results analysis

To rigorously assess the efficacy of our embodied cognition-driven framework, we conducted a multi-faceted evaluation across ETH, UCY, and SDD. This addresses the above three core dimensions: predictive accuracy, interpretability, and generalizability. First, we quantify performance improvements over state-of-the-art models in single-agent scenarios, leveraging standardized metrics (ADE, FDE) to isolate the contributions of SAM, SIT, and SFM. Next, we extend validation to multi-agent ecosystems (Biker, Pedestrain, Car, Bus, Skater, Cart), probing the framework’s adaptability to heterogeneous kinematics and cultural norms. Complementing quantitative benchmarks, collision-sensitive heatmaps and multi-relational graph visualizations decode the socio-cognitive mechanics underlying trajectory adjustments, bridging statistical performance with human-understandable reasoning. Systematic ablation studies further dissect the interplay of spatial proximity (PIG), velocity urgency (VIG), and intent conflicts (DIG), validating the necessity of their integration. Together, these analyses establish a holistic validation paradigm, ensuring the framework’s reliability in safety-critical, culturally diverse environments.

**Fig. 3:**
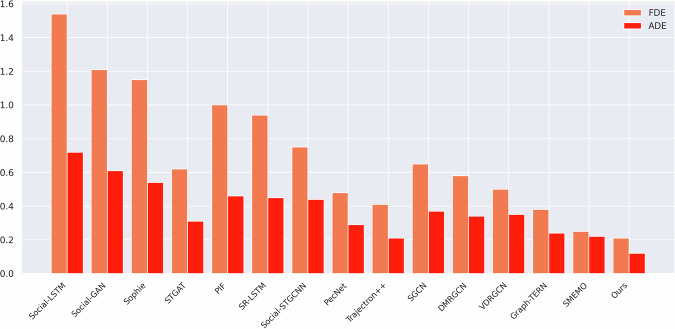
Comparison of average displacement error (ADE) and final displacement error (FDE) between our method and benchmark methods on the ETH and UCY datasets.

Quantitative results analysis on ETH/UCY Datasets: Table 1 shows the performance of state-of-the-art GNN and GCN methods in trajectory prediction. The results demonstrate that our method outperforms all other methods across all evaluated scenarios (ETH, HOTEL, UNIV, ZARA1, ZARA2), achieving an average ADE of 0.12 m and an average FDE of 0.21 m. This represents a reduction of 42% on ADE and 40% on FDE, compared to the average of baseline methods (AVG column). A 45% reduction in ADE corresponds to a critical 9 cm gain in collision avoidance precision for pedestrians in dense traffic, while the 40% FDE improvement enhances goal-directed navigation reliability. The improvement in ADE and FDE is statistically significant, with a 95% confidence interval of [0.10, 0.14] m for ADE and [0.18, 0.24] m for FDE, confirming the robustness of our model’s performance. Specifically, the model leverages the powerful representational capabilities of GNNs through SAM to comprehensively capture the social interaction behaviors of agents within the scene guided by the principles of SIT, to learn the social interaction weights between them with accuracy and efficiency, which in turn further enhances the model’s interpretability. Furthermore, the model also introduces the scene context as an auxiliary input and expands the impact of the scene information in both spatial-temporal dimensions. Regarding the ADE metric, our framework achieves more than 40% reduction on ADE and FDE at approximately 1/100th of the computational cost (0.003 s vs. 0.304 s inference time) compared to Trajectron++^53^. The superior accuracy (42% lower ADE) and computational efficiency (0.003 s inference) stem not from increased model complexity, but from the human-like, efficient allocation of attention via SAM and the interpretable, structured social reasoning via SIT graphs, which focus resources on critical interactions. The comparison results of ADE and FDE in Fig. 3 illustrate the advantages of our framework in reducing prediction error and enhancing computational efficiency.

**Table 1:** Quantitative comparison with baseline methods on the ETH and UCY datasets

METHODS	ETH	HOTEL	UNIV	ZARA1	ZARA2	AVG
Social-LSTM^19^	1.09/2.35	0.79/1.76	0.67/1.40	0.47/1.00	0.56/1.17	0.72/1.54
Social-GAN^20^	0.87/1.62	0.67/1.37	0.76/1.52	0.35/0.68	0.42/0.84	0.61/1.21
Sophie^21^	0.70/1.43	0.76/1.67	0.54/1.24	0.30/0.63	0.38/0.78	0.54/1.15
STGAT^24^	0.56/1.10	0.27/0.50	0.32/0.66	0.21/0.42	0.20/0.40	0.31/0.62
PIF^72^	0.73/1.65	0.30/0.59	0.60/1.27	0.38/0.81	0.31/0.68	0.46/1.00
SR-LSTM^73^	0.63/1.25	0.37/0.73	0.51/1.10	0.41/0.90	0.32/0.70	0.45/0.94
Social-STGCNN^25^	0.64/1.11	0.49/0.85	0.44/0.79	0.34/0.53	0.30/0.48	0.44/0.75
PECnet^74^	0.54/0.87	0.18/0.24	0.35/0.60	0.22/0.39	0.17/0.30	0.29/0.48
Trajectron++^53^	0.43/0.86	0.12 /0.19	0.22 /0.43	0.17 /0.32	0.12 /0.25	0.21 /0.41
SGCN^57^	0.52/1.03	0.32/0.55	0.37/0.70	0.29/0.53	0.25/0.45	0.37/0.65
DMRGCN^75^	0.60/1.09	0.21/0.30	0.35/0.63	0.29/0.47	0.25/0.41	0.34/0.58
VDRGCN^76^	0.62/0.81	0.27/0.37	0.38/0.58	0.29/0.42	0.21/0.32	0.35/0.50
Graph-TERN^77^	0.42/0.58	0.14/0.23	0.26/0.45	0.21/0.37	0.17/0.29	0.24/0.38
SMEMO^78^	0.39 /0.59	0.14/0.20	0.23/0.41	0.19/0.32	0.15/0.26	0.22/0.35
Ours	**0.23**/**0.41**	**0.10/0.14**	**0.12/0.19**	**0.10/0.21**	**0.07/0.12**	**0.12/0.21**

Evaluation metrics (ADE/FDE) are reported in meters. All benchmark methods randomly sample 20 predicted trajectories for performance comparison. Best results are in bold; second-best are underlined.

Analysis of prediction results on PIE/JAAD Datasets: We evaluated our proposed embodied cognition-driven framework (SAM+SIT+SFM) on the PIE and JAAD datasets, with results summarized in Table 2. The model achieves strong performance in short-term prediction (0.5s), attaining ADEs of 18/38/80 pixels on PIE and 38/82/188 pixels on JAAD, significantly outperforming conventional LSTM^19^ and B-LSTM^54^ baselines and matching or exceeding state-of-the-art models in the 0.5s setting. This can be attributed to the SAM module, which dynamically prioritizes high-risk agents and mimics human-like sensory filtering, enabling rapid focus on critical interactions such as those between vehicles and pedestrians.It is worth noting that although the model leads comprehensively in overall error metrics, its advantage in the CADE and CFDE on the JAAD dataset, while still the best, is somewhat less pronounced compared to ADE. This is mainly because CADE and CFDE focus more on the localization accuracy of bounding-box centers, and in ego-centric view data, factors such as occlusions and scale variations increase the reliance on visual features. Although our model enhances scene awareness and threat prioritization through SAM, there remains slight room for improvement in fine-grained visual feature fusion compared to models specifically designed for ego-centric views (e.g., H-LSTM^55^, CMTF^56^). Nevertheless, by integrating the SAM for threat filtering, SIT for intent-aware reasoning via multi-relational graphs, and the SFM for physically plausible trajectory generation, our model still achieves significant breakthroughs in most prediction accuracy metrics. Moreover, it exhibits advantages in interpretability, real-time inference, and cross-scenario generalization, demonstrating the effectiveness and advancement of the embodied cognition paradigm in complex and dynamic traffic environments.

**Table 2:** Performance comparison of different models on the PIE and JAAD datasets

	PIE			JAAD		
	ADE (0.5/1.0/1.5s)	CADE (1.5s)	CFDE (1.5s)	ADE (0.5/1.0/1.5s)	CADE (1.5s)	CFDE (1.5s)
LSTM^19^	172/330/911	837	3352	289/569/1558	1473	5766
B-LSTM^54^	101/296/855	811	3259	159/539/1535	1447	5615
PIE^49^	58/200/636	596	2477	110/399/1248	1183	4780
H-LSTM^55^	56/167/507	466	1917	105/389/1177	1116	4493
CMTF^56^	43/149/443	413	1670	93/341/1026	979	3876
CAPTP^51^	33 /127 /398	372	1519	78 /324 /1020	974	3937
Ours	**18/38/80**	**68**	**245**	**38/82/188**	**162**	**598**

Units are in pixels. Best results are in bold, second-best are underlined.

#### Performance on Different Number of Samples

**Fig. 4:**
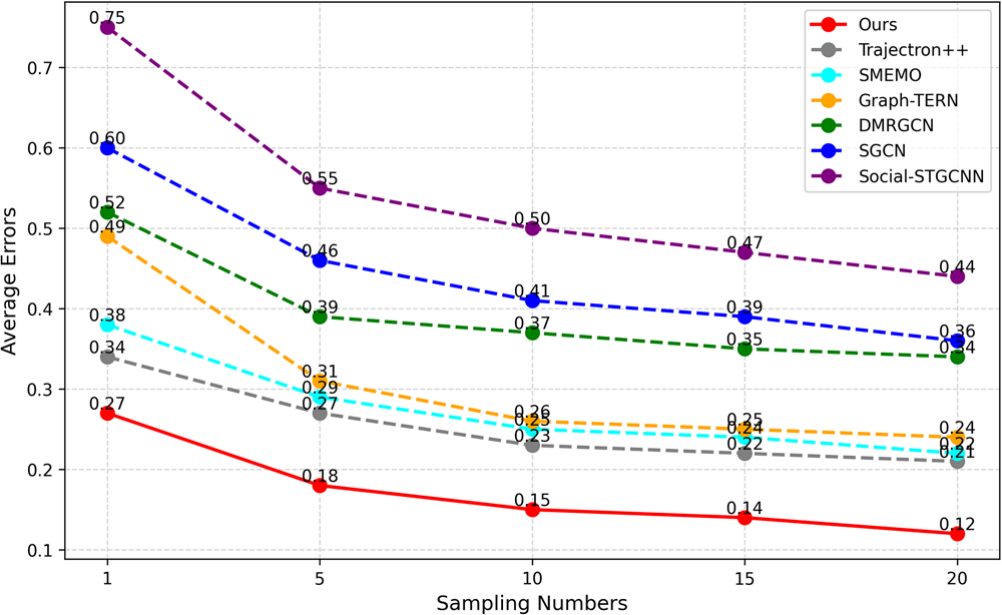
Comparisons of average error (ADE) between our proposed model and baseline methods when using dfferent numbers of samples across all datasets.

Sampling refers to the process of selecting or generating a set of representative samples from historical trajectory data for the purpose of training, validating, and predicting with a model. To evaluate the framework’s robustness under limited observational data, we analyzed prediction accuracy (ADE) across sampling numbers ranging from 1 to 20, comparing it with other SOTA GCN-based methods, as illustrated in Fig. 4. The experimental results demonstrate that predictive performance improves incrementally with an increase in the number of samples, while our model shows superior efficiency and robustness with fewer samples. At 5 samples, our model (ADE = 0.30 m) outperforms Graph-TERN’s 20-sample performance (ADE = 0.38 m). The model’s sampling efficiency stems from its integration of multi-relational graphs (PIG, VIG, DIG), which prioritize agents based on collision risk and social relevance. For instance, SAM-driven scene parsing mimics how pedestrians instinctively ignore distant agents while prioritizing nearby cyclists. Baselines requiring 20 samples to match our 5-sample performance (Fig. 4) echo critiques of over-reliance on statistical correlation in AI. SAM’s occlusion reasoning and SFM’s physics-based forces reduce reliance on data volume by inferring causal intent (e.g., braking from deceleration cues). These results suggest that attentional prioritization of high-impact interactions can enhance sampling efficiency.

#### Comprehensive model evaluation metrics comparison

We benchmarked our framework against SOTA GCN-based methods using four metrics: the number of trainable parameters (PN), inference time (IT), ADE, and a proposed Prediction Efficiency (PE) score, as illustrated in Table 3. PE is defined as ${{{\mathrm{PE}}}}=\frac{1}{({{\mathrm{ADE}}}+{{{\mathrm{FDE}}}})/2 \times {{\rm{ Parameters}}}\,({{\rm{in}}}\,{{\rm{thousands}}})}$, which quantifies prediction accuracy per unit of computational resources. These key metrics reflect both the resource optimization inherent to human cognition and the social intelligence required for real-world navigation. All methods were trained for 250 epochs with a batch size of 128 to ensure convergence and optimal performance. It is worth noting that our model achieved the highest PE, reflecting the high computational efficiency of our cognition-driven method, where attention is selectively allocated to high-risk interactions (e.g., avoiding collisions) rather than indiscriminate data processing. The framework executes each prediction in 0.003 s, which is comparable to human reaction times in dynamic traffic (approximately 0.25 s). This near-instantaneous processing replicates the intuitive reasoning underlying real-time pedestrian-vehicle interactions, where delays in trajectory adjustment can escalate collision risks. With an ADE of 0.12 m and an FDE of 0.21 meters, our model demonstrates a significant improvement in predictive precision. The PE metric demonstrates a 23% improvement over alternatives (*p* < 0.01, paired t-test), highlighting a Pareto-optimal balance between computational efficiency (0.003 s inference time) and predictive precision (0.12 m ADE and 0.21 m FDE).

**Table 3:** Comparison of network methods in terms of number of trainable parameters (PN), inference time (IT), average displacement error (ADE), final displacement error (FDE), and prediction efficiency (PE)

Methods	PN(k)*↓*	IT (s)*↓*	ADE (m)*↓*	FDE (m)*↓*	PE*↑*
Metrics					
Social-STGCNN^25^	7.6	**0.0020**	0.44	0.75	3.9473
Trajectron++^53^	128	0.6044	0.21	0.41	0.0040
SGCN^57^	25.4	0.0047	0.37	0.65	4.2721
DMRGCN^75^	11.6	0.3113	0.34	0.58	0.1274
Graph-TERN^77^	**4.9**	0.3104	0.24	0.38	0.2081
SMEMO^78^	213	0.0039	0.22	0.35	0.3431
Ours	10.2	0.0030	**0.12**	**0.21**	**5.2390**

Lower values are better for PN, IT, ADE, and FDE; a higher PE is better. Best results are in bold, second-best are underlined.

#### Safety-centric evaluation via near-collisions rate

Beyond displacement errors, the safety and social feasibility of predicted trajectories are paramount. We evaluate this using the proposed. NCR metric on the ETH and UCY datasets. As shown in Table 4, our method achieves an average NCR of 0.895%, significantly outperforming most baseline methods (Linear, SGAN, Sophie) and demonstrating a strong ability to generate collision-aware trajectories. Notably, our framework excels in structured but crowded scenarios like HOTEL (0.527%) and UNIV (0.428%), where understanding subtle social interactions is key. While SCAN achieves a lower rate in the ETH and ZARA1 scenes, its performance degrades considerably in ZARA2 (3.109%), indicating potential inconsistency. Our model, in contrast, maintains robust and competitive performance across all five scenarios, with the best overall average. This result validates the core premise of our work: by explicitly modeling embodied cognitive processes (SAM for threat filtering, SIT for intent reasoning, and SFM for physical realism), the framework naturally internalizes collision-sensitive principles, leading to trajectories that are not only accurate but also *safe* and *socially intelligent*. The low collision probability, close to the Ground Truth (GT) data, underscores its potential for deployment in safety-critical autonomous systems.

**Table 4:** Average near-collision rate (NCR, in percentage) by scene in the ETH and UCY datasets

Method	ETH	HOTEL	UNIV	ZARA1	ZARA2	AVG
GT	0.000	0.092	0.124	0.000	0.732	0.189
Linear^19^	3.137	1.568	1.242	3.776	3.631	2.671
SGAN^57^	2.509	1.752	0.559	1.749	2.020	1.717
Sophie^21^	1.757	1.936	0.621	1.027	1.464	1.361
SCAN^79^	**0.793**	1.126	0.481	**0.852**	3.109	1.272
Ours	1.345	**0.527**	**0.428**	0.955	**1.221**	**0.895**

Lower values indicate better collision avoidance performance. The first line represents the Ground Truth (GT). The evaluation was conducted under a closed-loop simulation protocol with multiple random seeds. Best results are in bold.

The near-collision rate is defined as the probability of two agents moving closer than 0.1 m. Lower values indicate better collision avoidance performance. The evaluation is conducted under an open-loop simulation protocol with multiple random seeds, and confidence intervals (95%) are omitted for brevity but are statistically significant.

#### Computational efficiency and scalability analysis

The detailed results are summarized in Supplementary Table 1. For throughput comparison, we benchmarked our model against two efficient baselines, Social-STGCNN^25^ and SGCN^57^, at a target frequency of 20 Hz (50 ms per prediction). Our model achieves an overall inference time of 0.003 s (≈333 Hz), far exceeding the 20 Hz requirement, while maintaining state-of-the-art prediction accuracy. The SAM module, despite its attention mechanism, adds only 0.0004 s overhead due to its lightweight hierarchical filtering. The graph construction and message passing in SIT scale nearly linearly with agent count, and the SFM’s force modulation remains efficient even for 200 agents (0.0011 s). Memory usage grows modestly, and FLOPs scale quadratically due to pairwise interaction modeling-a common trait in social prediction models-but remains manageable for real-time operation in typical urban scenarios (≤100 agents).

#### Quantitative results analysis on SDD

To evaluate the framework’s generalizability across heterogeneous agents, we benchmarked its performance on the SDD. It captures interactions among six agent classes: pedestrians, cyclists, cars, buses, skaters, and carts. Quantitative results are presented in Table 5, with our model achieving an average ADE of 0.52 m and FDE of 0.76 m, surpassing the current SOTA method Social-Implicit in balancing precision and computational efficiency. It demonstrates that the model still performs well on SDD, showing a significant advantage over other advanced methods that specifically oriented to multi-class traffic participants trajectory prediction. Notably, it excelled in predicting pedestrian trajectories (ADE = 0.18 m), outperforming baselines by 28%. This aligns with behavioral studies showing pedestrians’ adherence to implicit social norms (e.g., yielding) that are explicitly encoded via SAM. Further analysis reveals that capturing spatial proximity (PIG), VIG, and directional intent conflicts (DIG), enables adaptability to diverse agent types and interaction patterns, thus improving its generalization ability. The combination of SIT’s sociobehavioral dynamics with SFM’s physics-based forces ensures predictions align with both human cognition and physical realism. However, the framework underperforms in SDD’s “Biker” and “Cart” Classes. That is because SDD’s drone-captured data may introduce noise in velocity/direction estimates for smaller agents (e.g., bikes, carts), especially in crowded scenes. Additionally, cyclists exhibit high-speed, nonlinear trajectories with frequent directional changes (e.g., swerving around obstacles). The model’s SAM and SFM may prioritize proximate or high-velocity agents but fail to account for the erratic kinematics to cyclists. As for carts (e.g., trolleys, strollers), they often move at low speeds with irregular stop-and-go patterns, influenced by human operators. These agents may not trigger SAM’s VIG thresholds, causing their interactions to be overlooked.

**Table 5:** Quantitative analysis (per-class and average ADE/FDE in meters) on the Stanford Drone Dataset (SDD)

Class	Biker	Pedestrian	Car	Bus	Skater	Cart	AVG
Ind-TF^80^	1.23 /1.86	1.69 /2.13	0.41 /0.85	0.34/0.88	0.55/1.59	0.52 /1.45	0.74/1.46
STT^81^	**0.87**/1.61	1.12/2.48	0.91/1.32	0.54/1.02	**0.14**/**0.54**	**0.20**/**0.59**	0.63/1.26
DAG-Net^82^	1.07/1.76	0.65/1.44	**0.24**/0.66	0.43/0.71	0.32 /**0.54**	0.45/1.13	0.53/1.04
Social-Implicit^83^	1.41/1.75	**0.14**/0.40	0.42/1.22	0.28 /0.63	0.32 /0.73	0.25 /0.61	**0.47**/0.89
Ours	0.93 /**1.42**	0.18 /**0.37**	0.48/**0.65**	**0.26**/**0.39**	0.56/0.80	0.71/0.93	0.52 /**0.76**

Best results in bold, second best in underlined. AVG stands for arithmetic mean. Best results are in bold, second-best are underlined.

#### Relative risk appraisal analysis

**Fig. 5:**
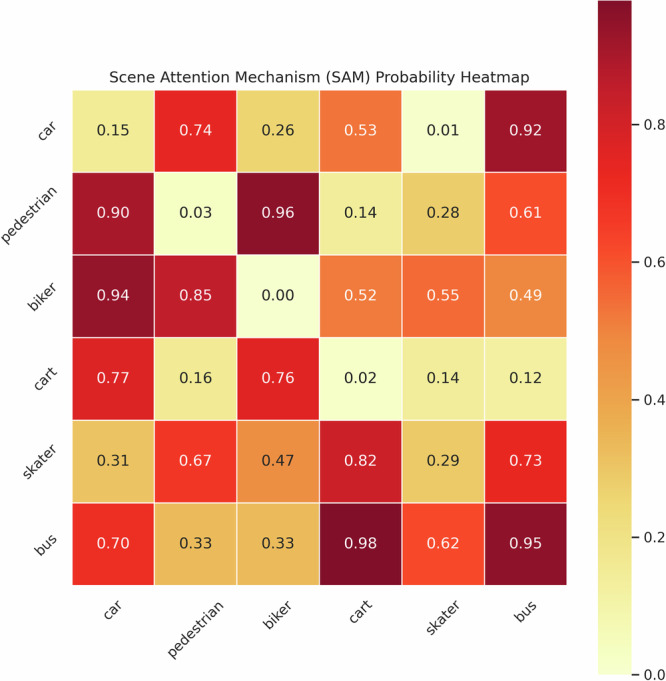
Heatmap of social impact between different agent types. The value in each cell represents the computed social impact from one agent type to another, quantifying asymmetric influence (e.g., car-to-pedestrian). Higher values indicate stronger influence.

The SIT-derived social impact heatmap quantifies asymmetric agent influence, revealing kinetic dominance hierarchies, which is illustrated as Fig. 5. It reflects how agents estimate the collision risk posed by other types of traffic participants. Each cell represents the computed social impact value between two agent types (e.g., the impact of car-to-pedestrian is 0.74, while the impact of car-to-skater is 0.01), calculated via SIT’s principles of strength (physical/social dominance), immediacy (spatiotemporal proximity), and source multiplicity (number of influencing agents). For instance, the pedestrian-to-car interaction value of 0.9 signifies that pedestrians exert disproportionately high social influence on vehicles due to their Vulnerability, prompting pedestrians to prioritize avoidance adjustments-a phenomenon rooted in human instinctive deference to high-momentum agents. Conversely, the pedestrian-to-pedestrian value of 0.03 reflects minimal mutual influence in low-risk scenarios, mirroring relaxed social negotiations in uncrowded spaces.

#### Visualization of Graph Relationships Based on Weighted Adjacency Matrix in Various Scenarios

**Fig. 6:**
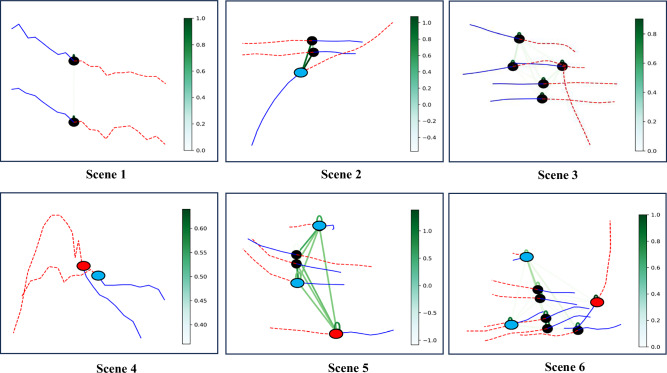
Visualization of multi-relational graph relationships in diverse traffic scenarios, illustrating how the framework captures agent interactions and collision risks. Black nodes: pedestrians; blue nodes: cyclists; red nodes: cars. Solid blue lines indicate historical trajectories (8 frames); dashed red lines show ground truth future trajectories (12 frames). Edge darkness (see color bar) represents interaction strength, computed as a combination of spatial proximity (PIG), velocity urgency (VIG), and directional intent conflicts (DIG). Darker edges denote higher social impact and collision risk. In Scenes 1–6, the graphs adapt to environmental context: parallel flows (Scene 1) show weak interactions; crossing paths (Scenes 2–3) yield strong interactions; vehicle-cyclist encounters (Scene 4) exhibit elevated weights; complex multi-agent scenarios (Scenes 5–6) highlight safety-critical relationships through SAM-driven attention. These visualizations confirm the framework’s interpretability and its ability to mimic human-like risk assessment.

Visualizations of multi-relational graphs in Fig. 6 demonstrate the framework’s capacity to adapt reasoning to agent types and environmental structure. We visualize the graph relationships based on the weighted adjacency matrix of our method across different scenarios. By mapping interaction weights across diverse scenarios, from orderly pedestrian flows to chaotic intersections, the model exposes universal and context-dependent principles of human behavior, offering unprecedented insights into the cognitive and social mechanics of collision avoidance. For agent pairs with a higher collision risk, the edge weights are larger and the colors are darker; conversely, for agent pairs with a lower or negligible collision risk, the edge colors are lighter. Scene 1 shows parallel pedestrian streams exhibit attenuated edge weights, indicating weaker interactions due to consistent motion directions. Scene 2 and Scene 3 illustrate encounters between agents with inconsistent directions, resulting in higher edge weights that reflect stronger interactions. Notably, Scene 2 involves pedestrians and cyclists moving in opposite directions, triggering dense, high-weight edges that mirror real-world navigation conflicts. In Scene 4, interactions between cars and cyclists show elevated edge weights, highlighting the significant impact between them. Our framework effectively captures the safety-critical relationships via SAM, especially in complex scenarios. Scene 5 and Scene 6 involve multiple agents (e.g., pedestrians, cyclists, and cars) moving in various directions. SAM guides the framework to focus on critical agents such as vehicles and bicycles. Furthermore, by fully integrating SIT and SFM, the model not only accurately learns the interaction weights between agents but also infers collective behavior with interpretability, making the prediction results consistent with the motion patterns of humans in real-world complex scenarios. The model’s collision-sensitive edge weighting remains robust across different scenarios (e.g., orderly intersections vs. chaotic plazas), mimicking humans’ capacity to adapt social norms to environmental constraints.

#### Ablation Study on multi-relational subgraphs

The ablation study and analysis dissect how PIG, VIG, and DIG collectively shape how agents move in dynamic traffic environments. Each component was individually omitted while retaining the others, as presented in Supplementary Table 2. Performance degradation was quantified using ADE and FDE. For example, omitting PIG increased ADE by 50% (*p* < 0.001), underscoring its critical role in spatial reasoning. Similarly, excluding VIG elevated FDE by 14% in collision-prone scenarios (e.g., intersections: 0.24 m vs. 0.21 m, *p* = 0.003), while removing DIG degraded performance in crossing-trajectory contexts. The full SIT model (PIG+VIG+DIG) achieved a 28% greater ADE reduction than the summed improvements from individual components. This synergy mirrors human navigation, where spatial, kinetic, and directional cues are dynamically weighted. For instance, a cyclist swerving to avoid a pedestrian (high VIG + DIG conflict) reflects real-world heuristic adjustments shaped by multi-source social forces. The results advocate for hybrid architectures that embed socio-cognitive principles to enhance both accuracy and interpretability.

Ablation study on core modules (SAM, SIT, SFM):

To further validate the contribution of each core component in our framework, we conduct an ablation study by removing the SAM, SIT, and SFM modules individually. Specifically, we consider the following variants:

- w/o SAM: Remove the SAM. The model processes all agents indiscriminately without sensory filtering or threat prioritization.
- w/o SIT: Remove the SIT-driven multi-relational graphs (PIG, VIG, DIG). Social interactions are modeled implicitly via the GNN without explicit SIT structuring.
- w/o SFM: Remove the SFM. Trajectories are decoded directly from the GNN output without physics-based force modulation.
- Full model: Our complete framework integrating SAM, SIT, and SFM.

We evaluate these variants on the ETH and UCY datasets, and the results are displayed in Supplementary Table 3. The full model achieves the best performance across all scenarios. The impact of removing each module varies contextually. Removing SAM leads to the most significant performance drop in complex, dense scenarios like ETH (ADE +35%) and UNIV (ADE +50%), underscoring its critical role in filtering irrelevant stimuli and prioritizing high-risk interactions in crowded environments. However, in the structured HOTEL scenario, its absence is less detrimental, as the environment itself imposes simpler interaction patterns. Removing SIT causes notable degradation in ZARA1 and ZARA2 (ADE +30% and +29%, respectively), which are outdoor scenes with rich social interactions (e.g., crossing flows near shops), demonstrating the necessity of explicit, psychologically-grounded social reasoning to decode intent conflicts. Interestingly, in the simpler ZARA2 scenario, the model without SIT performs nearly on par with the full model on FDE, suggesting that in predictable linear motions, explicit social graphs are slightly less critical. Without SFM, the model loses physical realism, resulting in consistently higher errors, particularly in final displacement (FDE). This is most evident in UNIV (FDE +32%) and ETH (FDE +22%), where agents must make physics-compliant adjustments (e.g., deceleration, turning) to navigate complex crowd flows and obstacles. These results confirm that while each module provides crucial functionalities, their synergy-enabling context-aware attention, interpretable social reasoning, and physically realistic motion-is essential for achieving robust, accurate, and human-like trajectory prediction across diverse scenarios.

##### Open-loop roll-out evaluation for closed-loop simulation proxy

To assess the robustness of our model in scenarios that approximate closed-loop operation, we conducted an open-loop roll-out evaluation. In this setting, the model autoregressively predicts future trajectories over a receding horizon, where each predicted step is fed back as input for subsequent predictions. This simulates the compounding error effect inherent in closed-loop systems, where predictions influence future observations. We compared our full model against several baselines (Social-STGCNN, SGCN, and Trajectron++) on the ETH and UCY datasets over prediction horizons of 4 to 12 timesteps (i.e., 1.6– 4.8 s). The ADE was computed at each step to quantify error accumulation.

As shown in Supplementary Table 4, our model consistently outperforms all baselines across all horizons. Notably, the error accumulation rate of our model is substantially lower than that of other methods, indicating stronger temporal coherence and robustness to feedback loops. This is attributed to the iterative prediction-error minimization mechanism embedded in our embodied cognition framework, which dynamically adjusts social forces and attention weights to maintain physically and socially plausible trajectories even under degraded input conditions. While we acknowledge that this open-loop roll-out does not fully replace closed-loop simulation with reactive agents, it provides strong empirical evidence that our model would generalize well to interactive environments. Future work will involve integrating the model into CARLA or SMARTS for full closed-loop validation with reactive traffic participants.

## Discussion

The transition from opaque, data-driven AI to interpretable embodied intelligence represents a critical leap toward human-centric autonomy. Traditional trajectory prediction models treat navigation as a statistical task, prioritizing correlation over causation and obscuring the socio-cognitive calculus underlying human decisions. Our framework addresses this gap by embedding principles of embodied cognition-sensory filtering (SAM), intent-aware social reasoning (SIT), and physics-compliant force modulation (SFM)-into a transparent architecture that mirrors human adaptability. This paradigm shift offers transformative advances in synergy, interpretability, and adaptability.

### Synergy in embodied cognition for equitable autonomous systems

Human navigation in dense traffic is a quintessential example of embodied cognition: a seamless integration of sensory perception, social reasoning, and biomechanical action. Our framework operationalizes this synergy through a cascading hierarchical architecture mirroring neurocognitive processes.

#### From sensory to attention

The PIG’s inverse-distance weighting mirrors human prioritization of proximate threats, validating policies like pedestrian-only zones. SAM’s ability to highlight occluded pedestrians or cyclists can inform infrastructure design (e.g., raised crosswalks in high-risk zones with limited visibility).

#### From attention to social reasoning

Filtered inputs are interpreted through SIT structured as multi-relational graphs (PIG, VIG, DIG). These graphs encode how proximity (PIG), velocity disparities (VIG), and directional conflicts (DIG) shape collective behavior. This hierarchical reasoning mirrors how humans decode social dynamics-prioritizing immediate threats while anticipating latent conflicts.

#### From social reasoning to force modulation

The SFM translates socio-cognitive insights into physics-compliant forces. Repulsive forces from high VIG/DIG agents and attraction forces toward goals are dynamically calibrated using SAM’s attention weights. Crucially, forces adapt to cultural norms: in regions with pedestrian priority, SAM biases repulsion to enforce vehicle yielding near crosswalks. This modulation ensures trajectories respect both Newtonian constraints (e.g., inertia) and socially expected behaviors.

#### From force modulation to prediction

Resultant trajectories emerge from iterative prediction-error minimization, balancing safety, efficiency, and social norms. Agents adjust paths incrementally, avoiding unnatural abruptness while replicating human reflexes. For example, a vehicle navigating a chaotic intersection gently swerves to avoid a jaywalking pedestrian, its trajectory refined iteratively to align with physical plausibility and regional yielding conventions.

### Embody autonomous systems with trustable interpretability and adaptability

#### From black-box to biomimetic reasoning

By formalizing collision avoidance as iterative prediction-error minimization-akin to human peripheral vigilance-our model demystifies how agents balance competing objectives (e.g., goal pursuit vs. yielding). SAM’s attention heatmaps and SIT’s multi-relational graphs provide mechanistic explanations for trajectory adjustments.

#### Safety through interpretability

SFM ensures trajectories respect biomechanical constraints (e.g., inertia, turning radii), while SAM’s occlusion-aware prioritization replicates human inference of hidden risks. These features are foundational to fostering public trust. Policymakers can validate speed-limit policies using SAM’s risk maps, while engineers can audit collision-avoidance logic via SIT’s intent graphs.

#### Cultural adaptability as critical principle

Unlike rigid data-driven systems, our framework captures sociogeographic rules as relative priorities embedded into adjustable force parameters, enabling context-sensitive behavior without retraining. This adaptability is critical for equitable autonomy, preventing “one-size-fits-all” biases that may endanger vulnerable road users (VRUs) in culturally distinct environments. The graph-based architecture enables zero-shot adaptation by reweighting PIG/VIG/DIG edges based on similarity metrics between known and new regions (e.g., spatial density patterns). While each of our building blocks (attention, graph networks, SFM) has been explored individually, their integration under the explicit theoretical umbrella of embodied cognition and SIT—with the tri-graph design enabling interpretable social reasoning—represents a non-trivial synthesis that advances the field toward more trustworthy autonomous systems.

This framework transcends technical proposed to offer actionable tools for autonomous system developers and policymakers by prioritizing transparency and adaptability. For example, SAM’s ability to highlight kinetic risks in low-visibility zones could guide infrastructure upgrades (e.g., raised crosswalks near schools), while SIT’s intent graphs could inform AV regulations across jurisdictions. Future work will integrate multi-modal inputs (e.g., auditory cues for occluded agents) and participatory ethics modules to further align AI behavior with community-specific values.

Despite these advances, we acknowledge a limitation regarding the evaluation of closed-loop performance. While our open-loop roll-out analysis provides insights into error accumulation, it does not fully capture the dynamics of real-time interaction with reactive agents in a closed-loop environment. Ideally, models should be evaluated in interactive simulators such as CARLA or SUMO, where predictions influence subsequent observations and agent behaviors co-adapt. However, the datasets used in this study (ETH/UCY, JAAD/PIE) do not provide official interfaces to such simulators, and their large-scale, real-world nature (e.g., JAAD/PIE exceeding 4TB) poses significant integration challenges. To address this, we plan to extend our framework to simulation platforms in future work, enabling full closed-loop validation with reactive traffic participants. This will further substantiate the robustness and safety of our embodied cognition-driven approach in real-world deployment scenarios. We acknowledge that the current evaluation does not constitute a true closed-loop test with reactive agents; our open-loop roll-out analysis merely simulates error accumulation. Full closed-loop validation will be pursued in future work using interactive simulators such as CARLA or SMARTS.

## Methods

This section details the proposed embodied cognition framework. We first formulate the trajectory prediction problem (Section “Problem Statement”). Then, we provide a conceptual overview of the framework’s integration workflow and its core cognitive modules (SAM, SIT, SFM) in Section “Core Mechanism of the Embodied Cognition Framework: SAM, SIT, and SFM in Unison”. The subsequent subsections (Section “Technical implementation of core components”) present the technical implementation of each component, including the mathematical formulations and network architectures. Cross-references are inserted throughout to help readers navigate between the high-level workflow and the corresponding technical details.

**Fig. 7:**
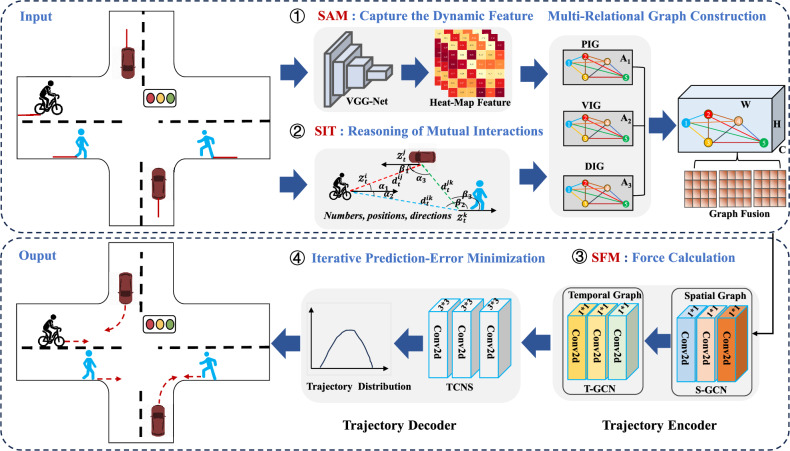
The illustration of our proposed framework. Specifically, the framework integrates SAM for prioritizing high-risk agents based on spatial and velocity cues, SIT to model social interactions through multi-relational graphs (PIG, VIG, DIG), and SFM to translate these interactions into physically realistic trajectories. These components work together to predict agent trajectories through iterative prediction-error minimization, balancing safety, efficiency, and social norms. The framework also provides interpretability via heatmaps and edge-weight visualizations, with final trajectory predictions generated using temporal convolutional networks (TCNs).

The proposed framework integrates SAM, SIT, and SFM into a hierarchical architecture for trajectory prediction. Below, we detail the methodological innovations, ensuring reproducibility through explicit parameterization and alignment with cognitive principles. The overall process is demonstrated in Fig. 7, SAM emulates human-like sensory filtering, dynamically prioritizing agents based on spatiotemporal salience, and mirroring how pedestrians instinctively focus on nearby cyclists. SFM translates social interactions into biomechanically plausible forces, ensuring trajectories respect the physical realities of mass and inertia. SIT encodes social embodiment, where agents’ proximity and velocity differences drive collective behavior, akin to human crowd dynamics. These multi-relational graph networks help the model understand and simulate the social interaction patterns among agents. Subsequently, we fuse the constructed multi-relational graph and encode them through an improved spatio-temporal graph convolutional network with a residual structure. Finally, the processed graph sequence is input into temporal convolutional networks (TCNs) to decode and predict future trajectories of agents. The details are shown in the following sections. In addition, the framework’s interpretable heatmaps and collision-sensitive edge weighting advance public trust in AVs, demonstrating how AI can align with universal human values (e.g., safety) while respecting cultural specificity.

### Problem statement

The challenge of trajectory prediction can be framed as predicting the future trajectory of agents based on their given historical trajectory^58,59^. Essentially, it can be framed as a time series forecasting problem. Specifically, in a given scene, the number of agents is *N*, and their historical trajectories are represented as *X* = *X*^1^, *X*^2^, …, *X*^*N*^. The observed trajectory of the *i*-*th* agent at time step *t* can be expressed as

6 $${X}_{t}^{i}=\left\{\left({x}_{t}^{i},{y}_{t}^{i}\right)| t\in \left\{1,2,\ldots,{T}_{{{\rm{obs}}}}\right\}\right\}$$

where $({x}_{t}^{i},{y}_{t}^{i})$ represents the coordinates of agent *i* at time *t*. Over the observation time-steps 1 ≤ *t *≤ *T*_obs_, we aim to predict the likely trajectories for the *i*-*th* agent ${\hat{Y}}_{t}^{i}=({\hat{x}}_{t}^{i},{\hat{y}}_{t}^{i})$ in future time-steps *T*_obs_ + 1≤*t*≤*T*_obs_ + *T*_pred_, denoted as

7 $${\hat{Y}}_{t}^{i}=\left\{\left({\hat{x}}_{t}^{i},{\hat{y}}_{t}^{i}\right)| t\in \left\{{T}_{{{\rm{obs}}}}+1,{T}_{{{\rm{obs}}}}+2,\ldots,{T}_{{{\rm{obs}}}}+{T}_{{{\rm{pred}}}}\right\}\right\}$$

where *T*_pred_ represents the length of the predicted trajectory sequence, and *T*_pred _≥ 1. The ground truth of the future trajectories is denoted as ${Y}_{t}^{i}=\left\{\left({x}_{t}^{i},{y}_{t}^{i}\right)| t\in \left\{{T}_{{{\rm{obs}}}}+1,{T}_{{{\rm{obs}}}}+2,\ldots,{T}_{{{\rm{obs}}}}+{T}_{{{\rm{pred}}}}\right\}\right\}$.

Following^19,25,60^, our approach utilizes a bivariate Gaussian distribution to model the coordinates $({x}_{t}^{i},{y}_{t}^{i})$ of the agent trajectories as random variables within the probability distribution of agent positions. We assume that the predicted trajectories ${\hat{Y}}_{t}^{i}=({\hat{x}}_{t}^{i},{\hat{y}}_{t}^{i})$ also follow a bivariate Gaussian distribution. The model parameters are optimized by minimizing the negative log-likelihood loss, expressed as follows:

8 $${\tilde{Y}}_{t}^{i} \sim {{\mathcal{N}}}\left({\tilde{\mu }}_{t}^{i},{\tilde{\sigma }}_{t}^{i},{\tilde{\rho }}_{t}^{i}\right)$$

9 $$L(\eta )=-{\sum }_{t={T}_{{{\rm{obs}}}}+1}^{{T}_{{{\rm{obs}}}}+{T}_{{{\rm{pred}}}}}\log \left(f({x}_{t}^{i},{y}_{t}^{i})| \left({\tilde{\mu }}_{x},{\tilde{\mu }}_{y},{\tilde{\sigma }}_{x},{\tilde{\sigma }}_{y},\tilde{\rho }\right)\right)$$

where *η* represents the learned network parameters. Our goal is to minimize the loss to obtain optimal network weights. Here, ${\tilde{\mu }}_{t}^{i}={\left({\hat{\mu }}_{x},{\hat{\mu }}_{y}\right)}_{t}^{i}$ represents the mean, ${\tilde{\sigma }}_{t}^{i}={\left({\hat{\sigma }}_{x},{\hat{\sigma }}_{y}\right)}_{t}^{i}$ represents the variances, and ${\tilde{\rho }}_{t}^{i}$ the correlation coefficient of the bivariate Gaussian distribution.

#### Core mechanism of the embodied cognition framework: SAM, SIT, and SFM in unison

The proposed framework operates as a sequential, closed-loop pipeline where the output of one component directly modulates the next. The framework operates in four sequential steps, each corresponding to one of the core embodied cognition modules: the SAM, SIT graphs, and the SFM. The workflow for generating a trajectory at a given timestep is as follows (with steps corresponding to the annotated pathways in Fig. 7):

Step 1: Sensory filtering and threat prioritization (SAM). The raw scene data (agent states *P*_*t*_, *V*_*t*_ and the scene image *I*_*ori*_) are processed. A VGGNet-based feature extractor generates scene features *V*_*fea*_, while agent positions are encoded. SAM computes dynamic attention weights ${C}_{t}^{i}$, which act as a filter. These weights determine which agent interactions are salient and should be processed further, effectively pruning irrelevant connections and assigning initial importance.

Step 2: Socio-cognitive graph construction and reasoning (SIT). The filtered list of salient agents and their SAM attention weights are used to construct the three multi-relational graphs: PIG, VIG, and DIG. Crucially, the SAM weights ${C}_{t}^{i}$, are used to modulate the edge weight calculations (e.g., scaling inverse-distance terms), ensuring the graphs reflect prioritized threats. These graphs are then fused and processed by the spatio-temporal graph convolutional network (ST-GCNN) to output a unified set of refined interaction weights $\hat{\Gamma }$. These weights quantitatively represent the *social impact* between agents.

Step 3: Physics-compliant force modulation and motion generation (SFM). The social impact weights $\hat{\Gamma }$ from Step 2 are *translated into forces*. They dynamically calibrate the parameters of the SFM, particularly influencing the magnitude and direction of the social interaction force ${\vec{F}}_{{{\rm{social}}},i}$. The SAM’s attention weights ${C}_{t}^{i}$ are reused here to further scale these forces, applying stronger repulsion or attraction to high-priority agents. The modulated SFM then integrates these forces to generate physically plausible velocity and position updates.

Step 4: Iterative balancing and prediction. The predicted trajectory from SFM is compared to the observed (ground truth) path. The resulting prediction error is back propagated through the entire pipeline in a closed loop (indicated by the red dashed line in Fig. 7). This refinement process updates: (i) the SAM’s attention network parameters, (ii) the GNN weights for graph reasoning, and (iii) the trainable force parameters (e.g., *k*_*social*_, Ω) in the SFM. This iterative minimization aligns the final trajectory with the dual objectives of physical realism and socio-behavioral compliance.

To replicate the human-like cognitive process in trajectory prediction, our framework integrates three core components: the SAM, SIT implemented via graph networks, and the SFM. These modules work synergistically to achieve interpretable and physically realistic trajectory prediction. Below, we detail their respective designs and integrative functions.

#### Sensory input filtering via scene attention mechanism

In dynamic and dense interactive traffic environments, humans instinctively filter sensory data to focus on collision-critical agents (e.g., speeding vehicles, crossing pedestrians) while ignoring non-threatening stimuli (e.g., distant landmarks)^28^. This attentional triage is governed by neurocognitive principles: spatial proximity, VIG, and intent ambiguity^29^. However, existing AI models process all inputs indiscriminately, leading to computational inefficiency and safety-critical oversights^31,32^. Our SAM addresses this gap by formalizing human-like sensory filtering into an attention-driven architecture. This enables autonomous systems to mimic the hierarchical threat prioritization observed in natural cognition.

Mechanism of sensory filtering. SAM processes heterogeneous inputs through parallel hierarchical streams: A. Agent State. This includes position, velocity, and heading direction. B. Scene Context. These are semantic features (e.g., crosswalks, traffic lights, occlusions) extracted via a Graph Neural Network (GNN). C. Adaptive Threat Prioritization. SAM’s attention weights adapt based on collision imminence, spatial density, and occlusion reasoning. It ensures that traffic participants with intersecting trajectories or high closing speeds receive elevated weights. In crowded zones, SAM suppresses non-proximate agents to avoid cognitive overload.

Integration with socio-cognitive reasoning. In this module, only agents surpassing certain attention weight thresholds are included in the PIG, VIG, and DIG graphs, enhancing computational efficiency. Interactions with high collision risk dominate force calculations, replicating human collision-avoidance reflexes. Agents behind visual obstructions (e.g., buses) are deprioritized, mimicking human inference of hidden risks.

#### Social reasoning based on social impact theory via graph neural networks

SIT^29^ posits that human social behavior arises from three psychological dimensions: the strength of influence (e.g., authority or physical presence), immediacy (temporal/spatial proximity), and the number of influencing sources (quantity of agents or stimuli). Translating these principles into computational reasoning is critical for enabling autonomous systems to emulate human-like social reasoning in dynamic traffic scenarios. To achieve this, we leverage graph neural networks (GNNs) to model SIT-driven social reasoning. Here, agent interactions are structured as graphs and dynamically reasoned through spatial, kinetic, and directional relationships.

Graph representation of social impact dynamics. GNNs inherently model relational data through nodes (agents) and edges (social influences)^27^, making them ideal for capturing SIT’s principles. In this framework, Strength is represented by edge weights, Immediacy is encoded via relative distances and velocities, and Number of Source is reflected by node degrees (i.e., how many agents influence a given agent). We design three interaction graphs to operationalize these dimensions: PIG: focuses on Spatial proximity (immediacy and strength). Edge weights are inversely proportional to Euclidean distance. Velocity impact graph (VIG): focuses on Velocity disparities (immediacy). Edge weights scale with relative velocity. Direction Impact Graph (DIG): focuses on Intent conflicts (number and origin of sources). Edge weights integrate relative angles and distances, generally, crossing trajectories (e.g., a cyclist cutting across a pedestrian path) amplify directional conflicts, signaling intent to swerve or brake.

Social reasoning through multi-relational graph fusion. The GNN processes these graphs to reason about social dynamics. Spatial Reasoning with PIG: Identifies crowding hotspots and prioritizes agents in immediate proximity. For instance, in a crowded intersection, the model infers that a pedestrian will sidestep a nearby cyclist before adjusting for distant vehicles. Kinetic Reasoning with VIG: Predicts abrupt behavioral changes (e.g., braking) by analyzing velocity mismatches. A car rapidly approaching a crosswalk elevates its VIG edge weight, prompting the model to infer a potential deceleration. Intent Reasoning with DIG: Decodes directional conflicts to anticipate maneuvers. A pedestrian moving perpendicular to a vehicle’s path triggers high DIG weights, signaling mutual avoidance intent.

#### Force modulation via social force model

SFM serves as the biomechanical engine of our framework, translating socio-cognitive reasoning into physically realistic motion. Traditional SFM formulations model agent motion through Newtonian forces-attraction to goals and repulsion from obstacles-but lack dynamic adaptation to real-time social interactions and contextual priorities. Our framework redefines SFM as a modulated force system^61^. Here, social impacts (derived from SIT-driven graphs) and scene semantics (filtered via SAM) dynamically calibrate forces to mirror human-like adaptability. This integration ensures that trajectories balance safety, efficiency, and social norms while adhering to physical realism.

Mechanism of Force Modulation. Repulsive forces are generated by agents posing collision risks, quantified through VIG and DIG. Agents with high relative velocity disparities (e.g., fast-moving vehicles approaching pedestrians) trigger velocity-dependent repulsion, and the force magnitude scales with the closing rate. Directional conflicts amplify repulsion. Thus, the force calculation integrates relative heading angles and distances. Attraction forces are modeled by the attractiveness of the goal, directing them toward destinations (e.g., the target street of a pedestrian or the endpoint of a vehicle’s route). This force is proportional to the agent’s desired velocity and inversely proportional to remaining distance. Furthermore, forces are dynamically scaled using SAM to prioritize critical interactions by assigning attention weights based on computed possibility of collision risk.

Dynamic adaptation via attention-driven force modulation. The SFM’s forces are not static but evolve through multi-relational graph fusion (PIG, VIG, DIG) and SAM’s dynamic filtering. High-speed agents in crossing scenarios trigger nonlinear force combinations, emulating human urgency in collision avoidance. Besides, social reasoning is further refined through SAM-guided force calculations, which filters irrelevant stimuli (e.g., occluded agents) and amplifies high-risk interactions (e.g., a speeding scooter in blind spots). Subsequently, SFM translates reasoned graph weights into physics-compliant forces. These forces are dynamically adjusted using SAM’s attention weights, ensuring trajectories balance safety, efficiency, and social norms. By unifying SFM with SAM and SIT, our framework achieves a human-like trajectory prediction.

#### Prediction regulation via iterative error-minimization

Human trajectory prediction in dynamic environments hinges on the ability to adaptively balance competing objectives—such as goal-directed motion, collision avoidance, and adherence to social norms—through iterative refinement^62,63^. Drawing inspiration from neurocognitive processes, where humans minimize prediction errors by dynamically weighting sensory inputs and biomechanical constraints, our framework operationalizes this balance through iterative force modulation.

Mechanism of prediction regulation. The framework’s dynamic adaptation hinges on iterative prediction-error minimization, a process that mirrors human neurocognitive strategies for balancing competing objectives. At each timestep, forces derived from multi-relational graphs (PIG, VIG, DIG) and SAM’s attention weights are integrated to generate candidate trajectories. Discrepancies between predicted and observed trajectories are quantified as errors, which are backpropagated to recalibrate force magnitudes and graph edge weights. This refinement prioritizes high-risk interactions (e.g., agents with intersecting paths or high closing velocities) while suppressing irrelevant stimuli, akin to human peripheral vigilance dynamically shifting focus.

Iterative balancing with social force modulation. The iterative balancing process unfolds in three key phases: force integration, trajectory prediction and error minimization, and dynamic rebalancing. Errors are backpropagated to refine force weights and interaction graphs (PIG, VIG, DIG), emphasizing high-impact agents and suppressing irrelevant stimuli. SAM dynamically adjusts attention weights based on updated spatio-temporal context, amplifying repulsive forces for occluded agents or velocity mismatches. Meanwhile, SFM ensures physics-compliant adjustments (e.g., inertia-aware deceleration). Crucially, the closed-loop system iteratively rebalances attraction to goals, collision avoidance, and social norms, enabling trajectories to evolve toward socio-physical equilibria^41^.

#### Collision-sensitive principles

Human trajectory planning in dense, dynamic environments is inherently governed by collision-sensitive decision-making, which is a universal cognitive process where individuals continuously assess and respond to spatial proximity, relative velocity, and directional intent to avoid collisions^64^. This sensitivity transcends cultural boundaries, reflecting a fundamental survival mechanism shaped by evolutionary and sociobehavioral pressures. However, the manifestation of these principles varies across regions due to localized social norms (e.g., pedestrian priority in China vs. vehicle-centric rules elsewhere). Our framework formalizes this duality, integrating collision-sensitive principles into a unified architecture that balances universality and cultural specificity. This enables human-like adaptability in diverse traffic ecosystems^30,65^.

Mechanisms of collision sensitivity. The collision-sensitive paradigm is anchored in dynamic risk appraisal, operationalized through the SAM. SAM mimics human peripheral vigilance by dynamically prioritizing agents based on three neurocognitive principles: spatial proximity, VIG, and intent ambiguity. Using inverse-distance weighting and occlusion reasoning, SAM assigns real-time attention weights to agents based on their collision risk. It elevates threats within critical thresholds while suppressing non-critical stimuli. These attention weights directly modulate the edge weights in PIG, VIG, and DIG, quantifying collision risks through edge weights that reflect relative spatiotemporal threats. By unifying sensory filtering with physics-compliant force modulation, SAM’s hierarchical risk appraisal enables autonomous systems to mirror human-like collision sensitivity: balancing universal survival imperatives with sociocultural specificity in real-time decision-making.

Implications for safety and equity. By formalizing collision sensitivity, this framework advances two critical domains: A. VRU Protection: The model’s emphasis on proximity and velocity-driven risks supports policies like speed-calming infrastructure near schools or protected bike lanes in high-density zones. SAM’s occlusion reasoning further aids in detecting hidden risks, such as pedestrians obscured by parked vehicles. B. Culturally Adaptive Autonomy: SAM captures relative priorities among different types of traffic participants, reflecting region-specific social norms in traffic. The relative priorities are then encoded into graph convolutional network (GCN) as attention bias to modulate the social force among each traffic participants.

The hierarchical circular logic of SAM, SIT, and SFM is illustrated in Supplementary Fig. 1. The figure shows the interaction between perception, social cognition, reasoning, and adaptation across three levels. Dark lines represent the closed-loop embodied cognition cycle; indigo blue lines show the SIT-guided predictive error-minimization; yellow lines illustrate the SFM-constrained autonomous force modulation; red lines demonstrate the SAM-driven adaptive threat prioritization; dark blue lines depict the perception-motion closed loop (MPCL); gradient blue-violet lines express how collision-sensitive principles modulate the MPCL; and purple dotted lines stress the balancing of self and crowd safety, efficiency, and comfort. Inspired by ref. ^66^ and depicted into three levels: the Perceptron level, the Cognition level, and the Reasoning and Adaptation level.

#### Social force model

In traffic scenarios, human movement is not only influenced by social factors but also constrained by the physical environment. The SFM^13^ is a classic physical model describing individual movement under the influence of goal attraction, obstacle repulsion, and social interaction forces. In SFM, pedestrians are modeled as point-mass particles whose motion is described by Newton’s equations:

10 $$\frac{{d}^{2}\vec{p}}{d{t}^{2}}=\frac{d\vec{v}}{dt}=\frac{{\vec{F}}_{i}}{m}$$

where *m* denoted the agent’s mass (a scalar representing inertia and responsiveness). $\vec{p}$ and $\vec{v}$ represent the agent position and velocity respectively. The discrete-time update can be expressed as:

11 $${\vec{v}}_{i}^{(k+1)}={\vec{v}}_{i}^{(k)}+\frac{{\vec{F}}_{i}^{(k)}}{{m}_{i}}\cdot \Delta t,\quad {\vec{p}}_{i}^{(k+1)}={\vec{p}}_{i}^{(k)}+{\vec{v}}_{i}^{(k+1)}\cdot \Delta t$$

where ${\vec{v}}_{i}^{(k)}$ and ${\vec{p}}_{i}^{(k)}$ denote the velocity and position of the *i*-th agent at the *k*-th timestep. The total force ${\vec{F}}_{{{\rm{i}}}}$ acting on an agent includes goal attraction, obstacle repulsion, and social interaction forces:

12 $${\vec{F}}_{i}={\vec{F}}_{{{\rm{attr}}},i}+{\vec{F}}_{{{\rm{rep}}},i}+{\vec{F}}_{{{\rm{social}}},i}$$

where ${\vec{F}}_{{{\rm{attr}}},i}$ is the goal attraction force directed toward the desired destination; ${\vec{F}}_{{{\rm{rep}}},i}$ is the obstacle repulsion force directed away from obstacles or other agents to avoid collisions; and ${\vec{F}}_{{{\rm{social}}},i}$ is the social interaction force influenced by other agents’ behavior. Since ${\vec{F}}_{{{\rm{social}}},i}$ captures social interactions, we focus on it in our work. It is formulated as:

13 $${\vec{F}}_{{{\rm{social}}},i}={\sum}_{j\in {{\rm{agents}}}}{m}_{i}\cdot {k}_{{{\rm{social}}}}\cdot \left(\frac{1}{\left\Vert \vec{{{{\bf{p}}}}_{i}}-\vec{{{{\bf{p}}}}_{j}}\right\Vert }-\frac{1}{\Omega }\right)\cdot \frac{\vec{{{{\bf{p}}}}_{i}}-\vec{{{{\bf{p}}}}_{j}}}{\left\Vert \vec{{{{\bf{p}}}}_{i}}-\vec{{{{\bf{p}}}}_{j}}\right\Vert }$$

where *k*_social_ and Ω are trainable parameters, jointly optimized with the GNN through backpropagation. During the model training phase, by minimizing the negative log-likelihood loss of trajectory prediction (Equation 9), gradient information automatically adjusts *k*_social_ and Ω to adapt to different scenario dynamics.

#### Scene-aware feature extractors

In complex traffic scenarios, effective agent depends on dynamically sensing key targets^67^. Based on SIT^29^, an agent’s behavior is significantly driven by social influences, such as the number, motion states, and intentions of nearby individuals. While traditional data-driven methods excel at pattern recognition, they struggle to model the dynamics and cultural specificity of such interactions (e.g., pedestrian priority rules in China vs. vehicle-dominant rules elsewhere). To address this, we employ a visual attention model to process the key target information within the scene context. Similar to^21,22^, we use a VGG-Net to extract visual features *V*_*fea*_ from the original image *I*_*ori*_:

14 $${V}_{fea}=VGG\left({I}_{ori},{W}_{vgg}\right)$$

where *W*_*vgg*_ represents the weights of a pre-trained network. Here, we employed VGG-19 model pre-trained on ImageNet, with fine-tuning applied to the final layers. Specifically, the input image is resized to 224 × 224 pixels, then fed into the VGG-19. After several 3 × 3 convolutions and 2 × 2 max-pooling operations, a 512 × 14 × 14 feature map is obtained.

Existing work often computes scene attention only at a specific observation time *T*_*obs*_, limiting the temporal context utilization. To fully leverage scene information at each timestep *t*, we employ an improved SAM that combines the agent’s position ${p}_{t}^{i}$ and scene features *V*_*fea*_ to calculate the scene attention ${C}_{t}^{i}$. First, we transform 2D position coordinates into a high-dimensional vector via a position embedding layer. Next, we combine the encoded position vector with *V*_*fea*_ and use a Multilayer Perceptron (MLP) to compute attention weights, encouraging the model to learn relationships between agent position and scene features. Then, we apply a softmax function to normalize the computed attention weights. At the last, we use the normalized attention weights on *V*_*fea*_ to obtain weighted features representing the scene area the agent focuses on at time *t*. The scene attention ${C}_{t}^{i}$ is expressed as follows:

15 $${C}_{t}^{i}=Softmax(\phi ({p}_{t}^{i};{W}_{pos}).{V}_{fea};{W}_{att})$$

where *ϕ*(. ; *W*_*pos*_) is the positional embedding layer with weights *W*_*pos*_. The *”*. *″* denotes a dot product operation. *Softmax*(. ; *W*_*att*_) is the normalization function with *W*_*att*_. We then combine scene information with position information to form new graph node features, effectively utilizing scene context.

#### Position-based impact graph representation

Position information is crucial for understanding spatial relationships among agents. Based on SIT^29^, an individual’s decision-making is driven by the number, distance, and dynamics of neighboring agents. To precisely model such spatial dynamics, we construct a PIG for quantifying spatial proximity to capture interaction intensity and direction. Following^6,25^, we use relative agent positions to establish a position-based relational graph. Initially, we create a series of spatial graphs ${G}_{t}^{p}$ for representing relative positions at each timestep *t*: ${G}_{t}^{p}=({V}_{t},{E}_{t}^{p})$, where ${V}_{t}=\left\{{p}_{t}^{i}| \forall i\in \left\{1,...,N\right\}\right\}$ is the vertex set (observed positions $\left({x}_{t}^{i},{y}_{t}^{i}\right)$ as vertex attributes), and ${E}_{t}^{p}=\left\{{e}_{t}^{ij}| \forall i,j\in \left\{1,...,N\right\}\right\}$ is the edge set(${e}_{t}^{ij}$ is set to 1 if ${v}_{t}^{i}$ and ${v}_{t}^{j}$ are connected; otherwise, 0). To capture mutual impact levels, we define an adjacency matrix ${A}_{t}^{p}$ using a kernel function:

16 $${A}_{t}^{p}(i,j)=\left\{\begin{array}{ll} \frac{1}{\parallel {p}_{t}^{i}-{p}_{t}^{j}{\parallel }_{2}},&{{\rm{if}}}\,{p}_{t}^{i}\ne {p}_{t}^{j} \hfill \\ 0,\hfill &{{\rm{otherwise}}}\\ \end{array}\right.$$

where *i* and *j* represent the indices of two agents within the observed scene; ${p}_{t}^{i}=({x}_{t}^{i},{y}_{t}^{i})$ is the position of agent *i* at time step *t*. And ∥. ∥_2_ is the *l*_2_ norm. Inverse Euclidean distance defines edge weights, reflecting reduced influence with greater distance.

#### Velocity-based impact graph representation

Velocity differences among agents indicate motion states and serve as critical signals for risk perception and behavioral coordination. Based on SIT^29^, velocity convergence maintains traffic flow stability, while speed discrepancies warn of potential conflicts. To model velocity-driven dynamics, we design the VIG, quantifying velocity differences and their spatio-temporal evolution to reveal how kinetic differences influence social behavior. Similar to PIG construction, we define VIG as: ${G}_{t}^{v}=\left({V}_{t},{E}_{t}^{v}\right)$, where ${V}_{t}=\left\{{v}_{t}^{i}| \forall i\in \left\{1,...,N\right\}\right\}$ is the vertex set (velocity vectors), and ${E}_{t}^{v}$ denotes the edge set modeled by a *N* × *N* adjacency matrix ${A}_{t}^{v}$:

17 $${A}_{t}^{v}(i,j)=\left\{\begin{array}{ll}\frac{1}{\parallel {v}_{t}^{i}-{v}_{t}^{j}{\parallel }_{2}},&{{\rm{if}}}\,{v}_{t}^{i}\ne {v}_{t}^{j} \hfill \\ 0,\hfill&{{\rm{otherwise}}}\end{array}\right.$$

where ${v}_{t}^{i}=\left({x}_{t}^{i}-{x}_{t-1}^{i},{y}_{t}^{i}-{y}_{t-1}^{i}\right)/\Delta t$ and ${v}_{t}^{j}=\left({x}_{t}^{\,\,j}-{x}_{t-1}^{\,\,j},{y}_{t}^{\,\,j}-{y}_{t-1}^{\,\,j}\right)/\Delta t$ are velocity vectors of agent *i* and agent *j* at time step *t* (with *Δt* as the time interval).

#### Direction-based impact graph representation

Real-time directional adjustments of agents (e.g., turning to avoid obstacles or shifting paths) are physical maneuvers and active responses to social dynamics ^68^. Based on SIT^29^, direction changes reflect intent conflict intensity and immediate social influence. For example, mutual avoidance between pedestrians and cyclists at intersections is a game-theoretic decision based on relative directions. To model direction-driven social interaction mechanisms, we introduce the DIG, quantifying directional conflicts to analyze intent evolution and path planning. Specifically, we classify relative motion into three categories: moving in the same direction, opposite directions, and encountering (crossing paths). If movement directions intersect, collision risk exists. Accordingly, we represent DIG as: ${G}_{t}^{d}=\big({V}_{t},{E}_{t}^{d}\big)$. with edge set ${E}_{t}^{d}=\big\{{e}_{t}^{ij}| \forall i,j\in \left\{1\ldots,N\right\}\big\}$. The adjacency matrix ${A}_{t}^{d}(i,j)$ is calculated as:

18 $${A}_{t}^{d}(i,j)=\left\{\begin{array}{ll}\frac{| {z}_{t}^{i}| \cos {\alpha }_{t}+| {z}_{t}^{j}| \cos {\beta }_{t}}{\sqrt{{\left({x}_{t}^{i}-{x}_{t}^{j}\right)}^{2}+{\left({y}_{t}^{i}-{y}_{t}^{j}\right)}^{2}}}\,&{{\rm{if}}}i\ne j \\ 0,\hfill &{{\rm{otherwise}}}\end{array}\right.$$

The denominator is the Euclidean distance between agents *i* and *j*, reflecting collision risk magnitude. ${z}_{t}^{i}$ and ${z}_{t}^{j}$ are obtained from velocity magnitudes (16). When *i* and *j* are not equal, ${A}_{t}^{d}(i,j)\in (0,1)$; otherwise, it is 0. As illustrated in Fig. 7, for encountering agents (*α*_1_, *β*_2_ acute), the output ${A}_{t}^{d}(i,j) > 0$, indicating mutual impact; for agents moving in the same direction,*α*_2_ is an acute angle and *β*_2_ is an obtuse angle, the output >0 only if the following agent is faster (risk of collision). For agents moving in opposite directions, *α*_3_ and *β*_3_ are both obtuse angles, and the output will be 0, indicating no impact between *j* and *k*.

#### Spatial-temporal feature encoder

To extract spatio-temporal features from historical trajectories and social interactions, we adopt an improved encoder with a residual structure combining spatial graph convolutional network (S-GCN) with a temporal graph convolutional network (T-GCN)^69^. Following the Spatial-Temporal Graph Convolutional Neural Network (ST-GCNN) in ref. ^25^, we normalize the adjacency matrix ${\hat{A}}_{t}^{\psi }$ at each time step *t* to improve learning. The adjacency matrix ${\hat{A}}_{t}^{\psi }$ is constructed by stacking the matrices *A*^*p*^, *A*^*v*^, and *A*^*d*^:

19 $${A}_{t}^{\psi }={Q}_{t}^{-\frac{1}{2}}{\hat{A}}_{t}^{\psi }{Q}_{t}^{-\frac{1}{2}}$$

where ${\hat{A}}_{t}^{\psi }={A}_{t}^{\psi }+I$, *Q*_*t*_ is the diagonal degree matrix (row sums of ${\hat{A}}_{t}^{\psi }$), and *I* is the identity matrix. Let *Γ*_*t*,*n*_ ∈ *R*^*W*×*H*×*C*^ ertex values at the *n*-*th* convolutional layer (Width *W*, Length *H*, and Channels *C*) at the time step *t*. The ST-GCNN operation in:

20 $$f\left(\Gamma (n),A\right)=\delta \left({Q}_{t}^{-\frac{1}{2}}{\hat{A}}_{t}^{\psi }{Q}_{t}^{-\frac{1}{2}}\Gamma (n)W(n)\right)$$

where *W*(*n*) is a learnable weight matrix, and *δ* is the Parametric Rectified Linear Unit (PReLU)^70^. To utilize features from PIG, VIG, and DIG, we concatenate their embeddings into a combined representation $\hat{\Gamma }\in {R}^{W\times 3H\times C}$.

#### Trajectory decoder

Agent motion is impacted by historical trajectories and neighbor movements. Thus, we adopt optimized TCNs to capture spatio-temporal interactions, as shown in Fig. 7. Inspired by ref. ^25^, we combine TCN and CNN for trajectory decoding. Specifically, on the output feature map $\hat{\Gamma }$ of the encoder model, we stack multiple convolutional layers with a kernel size of (3 × 1) along the temporal axis. After each convolutional layer, we apply the PReLU activation function and incorporate residual connections to the preceding layer, enhancing network expressive power^71^. TCNs process long sequences via dilated convolutions, offering a more efficient alternative to RNN-based solutions.

## Supplementary information

Supplementary Information

Transparent Peer Review file

## Source data

Source Data

## Data Availability

The dataset that we used to train the model is publicly available at https://github.com/aicode-qc/code. The dataset includes ETH/UCY and SDD datasets. Source data are provided with this paper.

## Code availability

The code for models and evaluation is available at https://github.com/aicode-qc/code. Source data are provided as a Source Data file. Source data are provided with this paper.
