# Supplementary material for: Embodied cognition-driven interpretable trajectory prediction of autonomous systems
Source: Nat Commun. 2026 Jul 6;17:8363. doi: 10.1038/s41467-026-75091-9 (PMC13473146; doi:10.1038/s41467-026-75091-9)
Supplement: Supplementary file 1 [file 41467_2026_75091_MOESM1_ESM.pdf]

Supplementary information:

Embodied Cognition-Driven Interpretable Trajectory Prediction of Autonomous Systems

Xiao Wang[1,2*], Quancheng Du[3], Qiong Wu[4,] Xiaofeng Jia[5],

Liang Lin[6], Ljubo Vlacic[7], Changyin Sun[1,8*], Fei-Yue Wang[9,10*]

1 Institute of Embodied Intelligence, Anhui University, Hefei, Anhui,230601, China.

2 Engineering Center of the Ministry of Education for Autonomous Unmanned Systems Technology, Anhui University, Hefei, Anhui, 230601, China.

3 School of Computer and Information Engineering, Henan Normal University, Xinxiang, Henan, 453007, China.

4 Anhui Jianghuai Automobile Group Corp., Ltd, Hefei, Anhui, 230008, China.

5 Beijing Big Data Center, Beijing, 100101, China.

6 School of Computer Science and Engineering, Sun Yat-sen University, Guangzhou, 510006, China.

7 Institute of Intelligent and Integrated Systems, Griffith University, Brisbane, 4222, Australia.

8 Anhui Province Engineering Center of Unmanned Systems and Intelligent Technology, Anhui University, Hefei, Anhui, 230601, China.

9 Faculty of Innovation Engineering, Macau University of Science and Technology, Macau, 999078, China.

10 DeSci Center of Parallel Intelligence, Obuda University, Budapest,H-1034, Hungary.

Supplementary Table 1

COMPUTATIONAL PROFILING AND SCALABILITY ANALYSIS OF THE PROPOSED FRAMEWORK FOR VARYING NUMBERS OF AGENTS (10, 50, 100, 200). METRICS INCLUDE TOTAL INFERENCE TIME, MODULE-WISE BREAKDOWN, MEMORY FOOTPRINT, FLOPS, AND THROUGHPUT.

Metric	10 Agents	50 Agents	100 Agents	200 Agents
**Total Inference Time (ms)**	1.82	2.51	3.00	4.20
SAM Attention (ms)	0.32	0.42	0.44	0.43
SIT Graph Build (ms)	0.42	0.60	0.81	1.20
SIT Message Passing (ms)	0.53	0.74	0.92	1.30
SFM Force Modulation (ms)	0.62	0.83	0.90	1.12
**Memory Footprint (MB)**	45	68	112	205
**FLOPs** (10^6)	12	85	280	950
**Throughput (Hz)**	≈555	≈400	≈333	≈238
**Throughput vs. 20 Hz Req.**	**27.8×**	**20.0×**	**16.7×**	**11.9×**
**(Social-STGCNN[25]) Time (ms)**	2.03	2.82	3.51	5.14
**(SGCN[57]) Time (ms)**	4.72	6.23	7.81	12.13

Note: All timings are averaged over 1000 runs. Throughput is calculated as 1 / Total Inference Time. Our model consistently outperforms both baselines in speed while maintaining higher accuracy (see Table 4). The SAM module introduces minimal overhead, and the framework scales gracefully to 200 agents while remaining well under the 50 ms (20 Hz) real-time threshold.

Supplementary Table 2

ABLATION STUDY ON THE CONTRIBUTION OF SPATIAL (PIG), VELOCITY (VIG), AND DIRECTIONAL (DIG) INTERACTION GRAPHS. RESULTS SHOW ADE/FDE (METERS) ON ETH/UCY SCENES. "√" MEANS INCLUDED; "×" MEANS OMITTED. BEST RESULTS ARE IN BOLD.

ID	PIG	VIG	DIG	ETH	HOTEL	UNIV	ZARA1	ZARA2	Average
1	×	√	√	0.49/0.78	0.08/0.12	0.12/0.21	0.12/0.22	0.08/0.13	0.18/0.29
2	√	×	√	0.39/0.64	**0.07/0.09**	**0.11**/0.19	0.11/0.17	0.09/**0.12**	0.15/0.24
3	√	√	×	0.42/0.69	**0.07**/0.10	**0.11**/0.19	0.12/0.20	0.08/0.13	0.16/0.26
4	×	√	×	0.43/0.71	0.09/0.13	0.14/0.22	0.15/0.21	0.11/0.14	0.18/0.28
5	√	×	×	0.47/0.72	0.08/0.12	0.12/**0.18**	**0.10/0.16**	0.09/0.13	0.17/0.26
6	√	√	√	**0.23/0.41**	0.10/0.14	0.12/0.19	**0.10**/0.21	**0.07/0.12**	**0.12/0.21**

Supplementary Table 3

ABLATION STUDY ON THE CORE MODULES (SAM, SIT, SFM). RESULTS SHOW ADE/FDE (METERS) ON ETH/UCY SCENES. BEST RESULTS ARE IN BOLD.

Model	ETH	HOTEL	UNIV	ZARA1	ZARA2	AVG
w/o SAM	0.31/0.55	0.11/0.16	0.18/0.30	0.13/0.25	0.08/0.13	0.16/0.28
w/o SIT	0.26/0.45	0.12/0.18	0.15/0.26	0.13/0.24	**0.07**/0.12	0.15/0.25
w/o SFM	0.28/0.50	**0.10**/0.17	0.16/0.28	0.14/0.26	0.09/0.15	0.16/0.27
Full model	**0.23/0.41**	**0.10/0.14**	**0.12/0.19**	**0.10/0.21**	**0.07/0.12**	**0.12/0.21**

Supplementary Table 4

Comparison of error accumulation (ADE, in meters) over increasing prediction horizons in open-loop roll-out evaluation on the ETH/UCY datasets. Lower values indicate better long-term stability.

Horizon (steps)	4	6	8	10	12
Social-STGCNN [25]	0.38	0.55	0.74	0.96	1.21
SGCN [57]	0.32	0.48	0.67	0.89	1.14
Trajectron++ [56]	0.21	0.33	0.48	0.66	0.87
Ours (Full Model)	**0.12**	**0.19**	**0.27**	**0.37**	**0.49**

Supplementary Figure 1

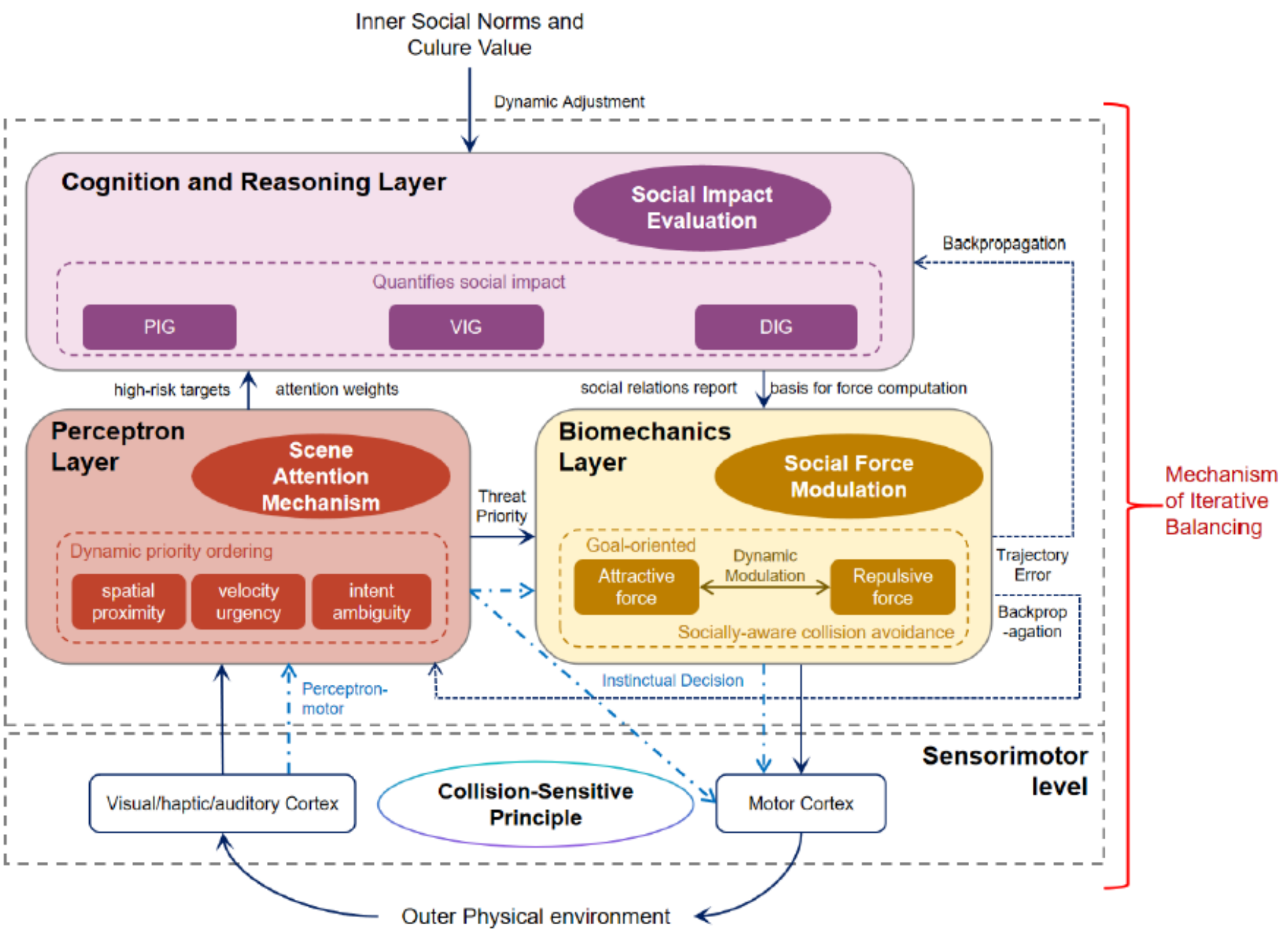

The hierarchical circular model of the embodied cognition framework.
