# Supplementary material for: Embodied cognition-driven interpretable trajectory prediction of autonomous systems
Source: Nat Commun. 2026 Jul 6;17:8363. doi: 10.1038/s41467-026-75091-9 (PMC13473146; doi:10.1038/s41467-026-75091-9)
Supplement: Supplementary file 2 [file 41467_2026_75091_MOESM2_ESM.pdf]

nature portfolio

Peer Review File

Embodied Cognition-Driven Interpretable Trajectory Prediction of Autonomous Systems

Corresponding Author: Professor Xiao Wang

This file contains all reviewer reports in order by version, followed by all author rebuttals in order by version.

Version 0:

Reviewer comments:

Reviewer #1

(Remarks to the Author)
This paper presents a cognitively inspired hierarchical framework for trajectory prediction of different agents in real-world scenes. The new methods proposed by the authors are relevant, and the discussions on the framework's capabilities and the parallels drawn with cognitive systems are meaningful. However, the structure and storyline of the paper need improvements to enhance clarity and coherence. Below are the detailed comments and suggestions for improvement.

Comments:
- Fig 2 is super dense: consider simplifying it, breaking it down for clarity, or reducing the text in it.
- The paper's structure and storyline are confusing, as the methods section comes after the results. The results section should be after the methods section, otherwise the reader cannot understand what methods were used to obtain the results. Thus, I suggest placing the results section after the methods section, and the discussion section after the results.
- The structure of the introduction can also be improved, and the analysis of the related literature is sparse in different parts of the introduction. Considering using a subsection named "Contributions", in which you concisely outline the contributions (thus avoiding repetitions of the same contributions multiple times in the introduction), and a subsection named "Related Work", so that the reader can easily follow the flow.
- The literature review is extremely long, but many subsections of it do not discuss nor cite existing papers, but rather mention existing methods without clear connections to literature papers. I suggest shortening the literature review and discussing / citing more clearly the relevant papers in each subsection.
- Also, from page 5 to 8, it appears that the authors mix the literature review with the methodology descriptions, which is very confusing. Please discuss all the methods in the "Methods" section, and close the literature review before presenting the methods.
- At the end of the literature review, a dedicated subsection summarizing the limitations in the literature would be beneficial to further motivate the contributions of the paper.
- A proposer conclusion is missing, and the limitation of the proposed methods should be better addressed.

Minor comments:
- The title of section 4 needs to be capitalized.

(Remarks on code availability)
The availability of the code is appreciated, and the structure of the GitHub repository is clear.

Reviewer #2

(Remarks to the Author)
Summary: The paper addresses the problem of trajectory prediction by combining the cognition and AI together through Scene Attention Mechanism (SAM), Social Impact Theory (SIT) and Social Force Model (SFM). The results shows the increase in performance of ADE and FDE score on benchmark datasets.

Comments:
1. In the SAM, the authors have used the VGGNet-19, which is kind of old model and if compare it with the Transformer model (which has better generalization ability), do the authors compare the performance of SAM standalone in their method?

2. It would be good to see, if the authors have shown the results on other more diverse datasets like JAAD, PIE and TITAN dataset to see the robustness of the proposed method.
3. From the novelty point of view, I believe it has minimal novelty because the methods like SAM, SIT and SFM are already developed and the authors just combined them in one framework which looks like the problem of engineering.
4. Since the authors have used the SFM, which has certain limitations like, needs parameters tuning, and difficulty with specific flow rates. How the authors addressed them?
5. The authors should provide the ablation study for their method, like removing or adding specific components in their method to see the effect of each one.

(Remarks on code availability)
The code looks fine to me. Have proper documentation.

Reviewer #3

(Remarks to the Author)
Summary: The paper proposes an “embodied-cognition” trajectory-prediction framework that chains (i) attention-based scene “sensory filtering,” (ii) Social Impact Theory–based social graphs, and (iii) Social Force–style biomechanics. The direction is interesting and interpretability-oriented, but the novelty is overstated, evaluation is limited (open-loop only), and the practical/real-world impact is unproven.

Pros:

- Clear conceptual pipeline mapping perception → social reasoning → physics.
- Interpretability motivation (attention, impact graphs, forces) is appealing.
- Claims strong ADE/FDE and fast runtime (if verified under matched protocols).

Cons:

- “First to unify” claim is not well supported by related work.
- Only open-loop evaluation; no closed-loop interaction safety/realism evidence.
- No stratified or robustness results for variable traffic or partial observability.
- Complexity/overhead and scalability are not characterized; reproducibility unclear.

More comments (first person):

1. **My biggest concern — open-loop evaluation only.** I see only open-loop results against logs. For an interaction-centric claim, I need **closed-loop** rollouts with collision/off-road rates, minimum inter-agent distance, time-to-collision, and smoothness/jerk, plus multi-seed CIs and head-to-head baselines under identical rollout protocols.
2. **Novelty/positioning.** I find the “first to unify sensory filtering + social cognition + biomechanics” claim overstated. Please either soften it or defend it with **direct, controlled** comparisons to prior social-attention and physics/force-aware graph models, clarifying what’s uniquely new (e.g., your SIT instantiation or tri-graph design).
3. **Variable traffic regimes.** I think it’s important to include results stratified by **low/med/high density**, **simple vs. conflict-prone scenes**, and **homogeneous vs. mixed agents** (peds/cyclists/vehicles, if relevant), with interaction metrics alongside ADE/FDE.
4. **Partial observability & perception noise.** To show the effectiveness, the paper should include robustness tests that are missing under **occlusions, dropped/late tracks, FOV limits, and noisy detections**. Please show degradation curves and explain how SAM/SIT/SFM behave when influential neighbors are missing or uncertain.
5. **Ablations & attribution.**

Ablation studies are also needed—replace/remove **SAM**, **SIT**, **SFM**, and each graph type—run **both open-loop and closed-loop** to show which components actually prevent drift/collisions and where the gains come from.

6. **Complexity, overhead, scalability.** Please profile **per-module latency** (attention, graph build/message passing, forces), **memory/FLOPs**, and scaling with **agent count** (e.g., 10→200). I want throughput comparisons to lean baselines at **10–20 Hz** on the **same hardware**.
7. **Runtime claim details.** The **0.003 s** number needs context: hardware, batch size, input size, and whether it **includes** graph construction and force computation. Show baseline runtimes under identical conditions.
8. **Operational / real-world impact.** Beyond displacement errors, I’m looking for evidence that the method **reduces unsafe or unnatural interactions**: closed-loop case studies, counterfactuals (e.g., remove/re-weight a high-impact neighbor), and human- or rule-based social-compliance scores.

(Remarks on code availability)

Reviewer #4

(Remarks to the Author)
The paper presents a modular framework for trajectory prediction based on cognitive principles, with a focus on interpretability. In particular it integrates Scene Attention Mechanism (SAM), Social Impact Theory (SIT), and a Social Force

Model (SFM) within a hierarchical architecture for trajectory prediction. A focus of this is on human / pedestrian trajectories and social attributes influencing these trajectories. The framework is evaluated on the ETH Walking Pedestrians Dataset, the UCY dataset, and the Stanford Drone Dataset (SDD), demonstrating superior results compared to related state-of-the-art approaches. Furthermore, the paper presents comprehensive experiments and an ablation study, providing insights on the role of the different modules.

Overall the paper presents an interesting approach combining different cognitively motivated methods to predict (human) trajectories, and showcasing good results. The presented methodology provides all necessary details and provides a sufficient understanding of the overall approach. However, there are a range of problems with the paper in the current form (see detailed comments), that I belief should be addressed. Most importantly, the organization of the paper lacks a concise structure, the Introduction introduces many concepts that are hard to relate to each other and choice to place the methodology after the results makes it hard to assess the evaluation completely. Furthermore, while the evaluation provides interesting insights and the authors present many details, the datasets are a bit outdated and it would be interesting to see how the presented approach performs on modern real-world datasets (see detailed comments).

Finally, the paper has quite a few typos and grammatical issues, that need to be fixed (see minor comments for some of them).

I therefore belief that the paper has to be revised substantially before it can be accepted.

Detailed Comments:
— The introduction is quite long and features a range of subsections and paragraphs, which are detailing a large range of foundational concepts of the proposed framework, both conceptually based on cognitive, social, neurological, etc. foundations, as well as the technical foundations. This makes it quite hard to parse and difficult to understand how these considerations are implemented / reflected in the framework. For the reader it would be very helpful to give more structure to this section, potentially also splitting it, and relate it more directly to the framework depicted in Fig. 2.

— The datasets used for evaluation are quite restricted and outdated, it would be interesting to understand how the approach would translate to more modern datasets, e.g., Pedestrian Situated Intent (PSI) [1]. This is in particular relevant, as the authors refer to predicting pedestrian trajectories in driving situations in the motivation, while all of the datasets used in the evaluation are based on top-down views and do not include the drivers / vehicle perspective.

— The structure of the paper is quite unconventional, as a reader I would expect to find the method (Section 3) before the results (Section 2). In the current way it is challenging to fully grasp the evaluation details, without having read the methodological details before.

— In section 3 the authors detail each component of the framework and explain the formal implementation of the components, however, for me it is not entirely clear, how these components are integrated within the framework. Fig. 8 provides a conceptual overview of the entire framework, but it stays at a rather high-level. It would be beneficial to provide a more detailed explanation on how the results of the different components plug together.

Additional comments:

— In the SDD (table 5) the biggest improvement seems to be with busses. What is the reason for this?

— The claim of enhancing computational efficiency is not really backed by a quantitative evaluation, it would be very helpful to add these results.

Minor:
Pg. 2 - cognitive processes, such as scene percepting —> perception

Pg. 3 - to model how human are impacted —> humans

Fig 2 appears very cluttered and is hard to parse. Several labels overlap with other elements. The upper part A is not clearly structured and the Cognition& Reasoning part is not very clear.

Pg. 3 - Specifically, this paradigm integrating embodied cognition and reasoning through: —> integrates

Pg. 3 - which mimicking human peripheral —> which is mimicking human peripheral

Pg. 3 - SFM modulates trajectories respect physical constraints —> SFM modulates trajectories to respect physical constraints

Pg. 6 - Attractive forces are modeled by —> Attraction

Table 3 - Authors are explaining the evaluation metrics in the text, however, it would increase readability if abbreviations would also be stated in the table captions.

Table 4 - AVG in caption and Average in the table.

Fig. 3 - Green arrow seems to be misplaced.

Pg. 10 I assume the ETH dataset is referring to the ETH Walking Pedestrians Dataset

Pg. 18 - The whole process is showed in —> shown

Pg 18 - mirrors how pedestrians instinctively focus —> mirroring

Pg. 20 - $\vec{p}$ and $\vec{v}$ are represent the agent position —> $\vec{p}$ and $\vec{v}$ represent the agent position

Pg 25 - 4 discussion —> Discussion

---

[1] Tina Chen, Renran Tian, Yaobin Chen, Joshua E. Domeyer, Heishiro Toyoda, Rini Sherony, Taotao Jing, Zhengming Ding: PSI: A Pedestrian Behavior Dataset for Socially Intelligent Autonomous Car. CoRR abs/2112.02604 (2021)

(Remarks on code availability)
The provided code seems to include all necessary functions to train and evaluate the model on the reported datasets. It comes Mit a brief readme giving the necessary instructions for installing and running the code. Furthermore, it also comes with the datasets (without the images) used in the paper, namely ETH, UCY, and SDD.

Version 1:

Reviewer comments:

Reviewer #1

(Remarks to the Author)
The changes made by the authors are appreciated. Still, I recommend the following minor improvements to improve the quality of the paper:
- In table 6, the computational times should be reported in ms or microseconds, with more significant digits
- In table 4, to quantify the trade-off between model accuracy and complexity (n. of trainable parameters), the authors could consider using the Akaike's information criterion.
- It is hard to interpret the message you want to convey with Fig. 6. Please consider improving it, together with its caption.
- in table 3, consider underlying the second-best results, for coherence with the other tables.
- always use a space between the text and references. In some parts of the text, this does not happen: for example, in the introduction, "environmental context to infer likely actions and conflicts[1, 2]" --> no space between conflicts and [1,2]. Same in many other parts.
- Minor errors remain to be fixed --> e.g. in the introduction "the gait of a pedestrian), ," --> two commas

(Remarks on code availability)
I appreciate the availability of the code, which seems sufficiently well documented.

Reviewer #2

(Remarks to the Author)
The authors have addressed all my concerns.
I have no further comments.

(Remarks on code availability)
The code looks good to me and now have proper documentation.

Reviewer #3

(Remarks to the Author)
Thank you for the revision and the added safety analysis; however, my primary concerns remain not fully addressed.

Closed-loop evaluation (still missing). By closed-loop, I mean closed-loop simulation in an interactive environment (e.g., CARLA/SUMO) and/or real-world AV experiments, where the model is run in a receding-horizon loop and predictions/actions are fed back to update subsequent states/observations (with reactive agents or planner/controller-in-the-loop, so errors compound and interactions respond). The added NCR appears to be a post-hoc near-collision check on predicted trajectories from offline datasets, which does not demonstrate true closed-loop performance.

Novelty is limited and incremental. Integrating graph/attention-based social interaction modeling with physics/force priors is well-studied; the SIT tri-graph framing and SFM coupling currently read as an incremental integration/interpretability layer

unless supported by controlled ablations/direct comparisons demonstrating unique benefits beyond generic interaction-graph/attention and standard dynamics/force-aware baselines.

(Remarks on code availability)

Reviewer #4

(Remarks to the Author)
The authors addressed most of my points by major restructuring and elaboration of the article. In particular:

— Restructuring of the introduction section improved readability significantly and made the arguments much clearer.

— The Newley introduced subsection on contributions help to better parse the framework in its entirety.

— Evaluation on newly added real-world datasets (JAAD, PIE) support the claims of the authors that the proposed framework adapts to real-world trajectory prediction.

— The elaboration on the description of evaluation metrics provide the reader with valuable guidance in interpreting the results.

— The newly added ablation studies help in assessing the effectiveness of the different parts of the framework.

— Addition of the description of the stepwise process and the improvements on Fig. 7 (previously Fig. 8) and the newly added Fig. 8 help in better understanding how the overall framework functions.

However, especially in Sec. 3, there is still a need for improvement. While the additions to Section 3 clearly improved its readability it is still quite dense and feels repetitive in some parts. I belief it could benefit from a clearer structure, e.g., by cross referencing relevant parts, and by a brief description of its structure in the beginning, to guide the reader through this rather lengthy section. Furthermore, as the descriptions in Sec 3.2 and the stepwise process in Sec 3.3 is highly related, authors may want to think about the possibility to move Sec 3.3. to the beginning of Sec. 3.2 and cross reference the corresponding subsections in the descriptions of the steps.

It would also be good to have a clear indication and separation in Sec 3, of parts providing a more conceptual overview and those giving the technical details. For instance, Sec. 3.4 - 3.10 could be placed as sub subsections in a separate section on technical details.

____
Minor:

— In section 3.3 the abbreviations (SIT, SAM, SFM) in the step heading, are a bit confusing, I belief they refer to the key methods used in each step, but this is not entirely clear.

(Remarks on code availability)

Version 2:

Reviewer comments:

Reviewer #1

(Remarks to the Author)
The changes and improvements made by the authors are appreciated. In my opinion, the paper can now be considered ready for publication.

(Remarks on code availability)
The code repository is sufficiently clear and useful to reproduce the results.

Reviewer #3

(Remarks to the Author)
Thank you for the detailed response and the added roll-out analysis. However, my main concern about the closed-loop evaluation remains. The new analysis is still open-loop and does not amount to true closed-loop evaluation with reactive agents. This is also reflected in the manuscript itself: while the limitations section acknowledges that the evaluation is not truly closed-loop, NCR is still described as a “closed-loop safety metric,” Table 5 refers to a “closed-loop simulation protocol,” and the paper claims to “close the perception-cognition-action loop.” I therefore think these claims should be softened, and the added analysis should be framed as open-loop robustness or safety evaluation.

Regarding novelty, the response clarifies that the key contribution is the SIT-based PIG/VIG/DIG tri-graph design, and the added ablation supports the usefulness of these components. Still, this mostly strengthens the empirical support for the method rather than its claim to be fundamentally non-incremental. The overall framework still appears to be an integration of established components, with the main novelty lying in the interpretable SIT-based formulation. I therefore view the novelty as moderate.

(Remarks on code availability)

Open Access This Peer Review File is licensed under a Creative Commons Attribution 4.0 International License, which permits use, sharing, adaptation, distribution and reproduction in any medium or format, as long as you give appropriate credit to the original author(s) and the source, provide a link to the Creative Commons license, and indicate if changes were made.
In cases where reviewers are anonymous, credit should be given to 'Anonymous Referee' and the source.
The images or other third party material in this Peer Review File are included in the article's Creative Commons license, unless indicated otherwise in a credit line to the material. If material is not included in the article's Creative Commons license and your intended use is not permitted by statutory regulation or exceeds the permitted use, you will need to obtain permission directly from the copyright holder.
To view a copy of this license, visit https://creativecommons.org/licenses/by/4.0/

Authors' Reply for NCOMMS-25-46440

"Embodied Cognition-Driven Interpretable Trajectory Prediction of Autonomous Systems"

First, we sincerely thank the Editor-in-Chief, Associate Editor, and all reviewers for their constructive feedback, which has greatly improved our manuscript. We are pleased to provide a detailed point-by-point response below, outlining the specific revisions we have made in the updated manuscript, with all changes clearly marked in blue for your convenience.

Modification and Explanation for the Comments from Reviewer 1

This paper presents a cognitively inspired hierarchical framework for trajectory prediction of different agents in real-world scenes. The new methods proposed by the authors are relevant, and the discussions on the framework's capabilities and the parallels drawn with cognitive systems are meaningful. However, the structure and storyline of the paper need improvements to enhance clarity and coherence. Below are the detailed comments and suggestions for improvement.

We are truly thankful for your positive assessment of the relevance of our work and for your detailed suggestions, which have significantly enhanced the manuscript's clarity and narrative flow.

1. Fig 2 is super dense: consider simplifying it, breaking it down for clarity, or reducing the text in it.

Explanation and Modification:

Thank you for this helpful suggestion. We have completely redesigned Figure 2 to improve its readability. The revised figure features simplified text with more concise labels, an optimized layout that clearly aligns the human and autonomous system cognition processes for easier comparison, and the use of clearer icons and a color-coded legend to distinguish different information flows.

2. The paper's structure and storyline are confusing, as the methods section comes after the results. The results section should be after the methods section, otherwise the reader cannot understand what methods were used to obtain the results. Thus, I suggest placing the results section after the methods section, and the discussion section after the results.

Explanation and Modification:

We appreciate the reviewer's valid perspective and have fully addressed all other suggestions to enhance the manuscript's clarity and rigor. We respectfully note that the structure—Introduction, Results, Discussion, Methods—follows Nature Communications formatting guidelines, which prioritize findings upfront. We have

ensured the Results section is self-contained, with full methodological details provided in Section 3.

3. The structure of the introduction can also be improved, and the analysis of the related literature is sparse in different parts of the introduction. Considering using a subsection named "Contributions", in which you concisely outline the contributions (thus avoiding repetitions of the same contributions multiple times in the introduction), and a subsection named "Related Work", so that the reader can easily follow the flow.

Explanation and Modification:

We appreciate your thoughtful comments on the manuscript's flow. We agree that consolidating the contributions and providing a more focused discussion of prior work would significantly improve the flow and clarity for the reader.

In response to this valuable suggestion, we have thoroughly reorganized the Introduction section (Page 2) by introducing two new subsections:

1.1. Related Work: This new subsection provides a more coherent and focused overview of existing trajectory prediction methods, clearly highlighting their limitations in interpretability and socio-cognitive modeling, which sets the stage for our proposed framework.

1.4. Contributions: This new subsection succinctly and explicitly lists the key contributions of our work in a bulleted format. This effectively avoids the repetition of contributions throughout the introduction and provides the reader with a clear and immediate understanding of the paper's novel elements.

Additionally, to further enhance the clarity and coherence of the storyline as you suggested, we have thoroughly restructured the Introduction (Section 1). We have now strictly separated the literature review, limitations analysis, high-level framework overview, and contributions statement into distinct subsections. All detailed methodological descriptions of SAM, SIT, and SFM have been consolidated into the Methods section (Section 3). This revision ensures a logical flow from problem identification to our proposed solution, significantly improving readability. To ensure these key innovations are prominently and clearly communicated to the reader, we

have revised the "Contributions" subsection (1.4) in the manuscript. It now explicitly lists, among other points, the "first data-driven computational model of SIT" and the "first unified architecture closing the perception-cognition-action loop" as distinct contributions, directly addressing the limitations outlined in the new Section 1.2.

4. The literature review is extremely long, but many subsections of it do not discuss nor cite existing papers, but rather mention existing methods without clear connections to literature papers. I suggest shortening the literature review and discussing / citing more clearly the relevant papers in each subsection.

Explanation and Modification:

Thank you very much for these critical observations. We acknowledge that certain subsections were overly descriptive without establishing strong, citation-backed connections to the specific body of work they aim to discuss. In our revision, we will comprehensively address this concern by:

（1） Condensing the Review: We significantly shorten the overall section by removing general descriptions and consolidating overlapping discussions.

（2） Strengthening Citations & Dialogue: For every key point or method mentioned (e.g., limitations of data-driven models, principles of SFM or attention mechanisms), we ensure a direct and clear citation to the most relevant foundational and contemporary papers.

（3） Improving Logical Flow: We reorganize the subsections to create a more coherent narrative—tracing the evolution from classical models to recent data-driven and hybrid approaches—and explicitly state how each discussed thread of literature leads to the identified research gap that our work fills.

These changes will transform the literature review from a descriptive overview into a focused, critical analysis that firmly grounds our research motivation in the existing scholarly conversation. We believe this will greatly enhance the section's rigor and clarity.

5. Also, from page 5 to 8, it appears that the authors mix the literature review with the methodology descriptions, which is very confusing. Please discuss all the methods in the "Methods" section, and close the literature review before presenting the methods.

Explanation and Modification:

We would like to appreciate the reviewer for this insightful comment regarding the structural organization of our manuscript. We agree that the previous interleaving of methodological descriptions within the introductory and literature review sections (Pages 5-8) could disrupt the narrative flow and create confusion for the reader.

In direct response to this valuable feedback, we have thoroughly reorganized the manuscript to ensure a clear separation between the literature review and the methodology. Specifically:

1. We have closed the literature review within the Introduction section (Section 1). The Introduction now focuses on establishing the research gap and motivating our work, concluding with a concise summary of our contributions in the new "Contributions" subsection, without delving into the detailed mechanics of our proposed framework.

2. All detailed descriptions of our proposed methods have been consolidated into the dedicated Methods section (Section 4), as per the standard structure of “Nature Communications.” This includes the comprehensive explanations of the Scene Attention Mechanism (SAM), the Social Impact Theory (SIT)-driven graphs (PIG, VIG, DIG), and the Social Force Model (SFM) modulation, which were previously discussed in the early parts of the manuscript.

This restructuring ensures that the reader first gains a clear understanding of the problem context and related work in the Introduction, before encountering the full technical details in the Methods section. We believe this significantly improves the logical flow and readability of the paper.

6. At the end of the literature review, a dedicated subsection summarizing the limitations in the literature would be beneficial to further motivate the contributions of

the paper.

Explanation and Modification:

This is an excellent suggestion. We agree that a clear summary of the limitations in the existing literature will sharply define the research gap and provide a stronger motivation for our work. We have added a new dedicated subsection titled "**1.2 Limitations of Existing Approaches**" immediately following the literature review. This subsection explicitly lists the key shortcomings of current methods, which serves to sharply define the research gap and provides compelling motivation for our proposed framework.

7. A proposer conclusion is missing, and the limitation of the proposed methods should be better addressed.

Explanation and Modification:

Thanks very much for highlighting this. We agree that these elements are crucial for a comprehensive research paper. In response to your suggestions, we have added a new, standalone **Section 5: Conclusion**. This section summarizes our core contributions and findings, and includes a dedicated paragraph that thoughtfully discusses the limitations of our current framework and outlines clear directions for promising future research. We believe these additions significantly enhance the completeness and rigor of our paper. The specific modifications made to the manuscript are detailed below for your review.

8. The title of section 4 needs to be capitalized.

Explanation and Modification:

We sincerely appreciate your attention to details that made the manuscript more qualified for *Nature Communications*. The title of Section 4 has been corrected to “Discussion” to adhere to proper formatting conventions.

Modification and Explanation for the Comments from Reviewer 2

The paper addresses the problem of trajectory prediction by combining the cognition and AI together through Scene Attention Mechanism (SAM), Social Impact Theory (SIT) and Social Force Model (SFM). The results show the increase in performance of ADE and FDE score on benchmark datasets.

We are grateful for your recognition of our work's performance and for your incisive questions, which have allowed us to clarify and strengthen several important aspects of the manuscript.

1. In the SAM, the authors have used the VGGNet-19, which is kind of old model and if compare it with the Transformer model (which has better generalization ability), do the authors compare the performance of SAM standalone in their method?

Explanation and Modification:

Thank you for raising this important point regarding model choice. This is a valuable point that allows us to clarify our design rationale and the role of the visual backbone within our broader framework.

Rationale for VGGNet-19: The primary role of the visual feature extractor in SAM is to provide a stable and semantically rich representation of the static scene context (e.g., crosswalks, drivable areas, building layouts). For this specific purpose, well-established and computationally efficient architectures like VGGNet have been proven highly effective in numerous trajectory prediction studies. Our focus was not on pushing the boundaries of scene understanding models but on leveraging a reliable, well-understood backbone to demonstrate the core contribution of our work: the novel integration of scene semantics with embodied cognition principles (SIT and SFM) for Interpretable and socially-aware trajectory prediction.

We acknowledge the reviewer's valid point regarding the advanced capabilities of Transformer models. While our current implementation effectively meets its objectives, we agree that exploring modern vision backbones, including Vision Transformers, is a promising direction for future work. Such models could potentially capture more complex long-range dependencies in the scene, which might lead to further

improvements. We have added a sentence in the revised Discussion section (Page 26) to acknowledge this limitation and future direction.

2. It would be good to see, if the authors have shown the results on other more diverse datasets like JAAD, PIE and TITAN dataset to see the robustness of the proposed method.

Explanation and Modification:

We sincerely appreciate this constructive suggestion to further demonstrate robustness of our framework. In direct response to this valuable point, we have added experiments on the JAAD and PIE datasets in Section 2.4. This section presents quantitative results and analysis of our model's performance on the JAAD (Joint Attention in Autonomous Driving) and PIE (Pedestrian Intention Estimation) datasets. These datasets introduce significant additional complexity compared to ETH/UCY/SDD, featuring richer and more challenging scenarios such as strong vehicle-pedestrian interactions, diverse pedestrian intentions (e.g., crossing/not crossing), and ego-vehicle perspectives. The new results confirm that our embodied cognition-driven framework maintains strong performance and generalizability. On both JAAD and PIE, it achieves competitive results compared to state-of-the-art intention-aware prediction baselines. This extended validation strengthens our claim regarding the model's robustness and adaptability to diverse, real-world traffic environments.

3. From the novelty point of view, I believe it has minimal novelty because the methods like SAM, SIT and SFM are already developed and the authors just combined them in one framework which looks like the problem of engineering.

Explanation and Modification:

We sincerely thank the reviewer for raising this important point regarding the novelty of our work. We respectfully acknowledge the reviewer's perspective that

individual components such as attention mechanisms (SAM), Social Impact Theory (SIT), and Social Force Models (SFM) have been explored in prior literature. However, we would like to clarify that the core contribution of our paper lies not in the invention of these individual concepts, but in their novel computational synthesis under a unified embodied cognition framework—a synthesis that involves fundamental conceptual and methodological advances beyond a straightforward engineering combination.

Prior to our work, these elements—sensory filtering (SAM), socio-psychological reasoning (SIT), and physics-based motion (SFM)—were largely explored in isolation. Data-driven models lacked interpretability and socio-cognitive grounding, while classical models like SFM lacked dynamic, intent-aware reasoning. Our key contribution is the theoretical framing and computational synthesis of these components into a coherent, interpretable architecture that mirrors the hierarchical, closed-loop processes of human cognition (perception→cognition→action), as illustrated in Fig. 3 and Fig. 8.

Specifically, our work introduces two key innovations:

1. **First Data-Driven, Quantifiable Computational Model of Social Impact Theory (SIT)**: While SIT is a well‑established psychological theory, to the best of our knowledge, it has never before been formalized as a data-driven, quantifiable computational model for trajectory prediction. We have translated its abstract principles—strength, immediacy, and number of sources—into a dynamic multi-relational graph framework (PIG, VIG, DIG). This allows us to explicitly and interpretably model social influence in a way that is both grounded in cognitive theory and fully learnable from real-world interaction data.

2. **First Unified Architecture Closing the Perception–Cognition–Action Loop**: Prior works have typically advanced individual aspects (e.g., social attention or physics-based forces). Our framework is, to our knowledge, the first to integrate sensory filtering (SAM), intent-aware social reasoning (SIT), and physics-compliant biomechanics (SFM) into a single closed-loop, hierarchical architecture that mirrors the embodied cognitive processes of humans. This integration enables the model to dynamically balance competing objectives—safety, efficiency, and social norms—

through iterative prediction□error minimization, resulting in trajectories that are not only accurate but also interpretable and socially compliant..

Thus, our contribution transcends a mere “engineering” combination. It represents a paradigm shift from black-box pattern matching to a principled, interpretable, and cognitively-inspired approach for trajectory prediction. The significant performance gains (42% reduction in ADE) and the novel interpretability features (risk-sensitive heatmaps, multi-relational graphs) demonstrated in our experiments are direct outcomes of this innovative integration.

To ensure these key innovations are prominently and clearly communicated to the reader, we have revised the “Contributions” subsection (1.4) in the manuscript. It now explicitly lists, among other points, the “first data-driven computational model of SIT” and the “first unified architecture closing the perception-cognition-action loop” as distinct contributions, directly addressing the limitations outlined in the new Section 1.2.

We are happy to further clarify these points in the manuscript and thank the reviewer again for prompting this important discussion.

4. Since the authors have used the SFM, which has certain limitations like, needs parameters tuning, and difficulty with specific flow rates. How the authors addressed them?

Explanation and Modification:

We sincerely thank the reviewer for this insightful and important question regarding the limitations of the classical Social Force Model (SFM). We fully acknowledge that traditional SFM formulations often rely on hand-tuned parameters and struggle to adapt to diverse or dynamic flow rates. In our work, we have addressed these fundamental limitations through a principled, learning-based integration within our embodied cognition framework:

1. **Data-Driven Parameter Learning**: Instead of using fixed, empirically tuned parameters, we treat the core SFM parameters—most importantly, the social force coefficient k_{social} and the interaction range parameter Ω in Equation (13) (Section 3.3)—as trainable variables. These parameters are jointly optimized with the entire

Graph Neural Network via back‑propagation, by minimizing the trajectory prediction loss (Equation 9). This allows the model to automatically learn context□appropriate force magnitudes and interaction distances directly from real□world trajectory data, eliminating manual tuning and enabling adaptation to various interaction intensities and scene dynamics.

2. **Dynamic Force Modulation via SAM and SIT**: The forces generated by the SFM are not static. They are dynamically modulated in real□time by the attention weights from the Scene Attention Mechanism (SAM) and the edge weights from the SIT□driven graphs (PIG, VIG, DIG). This means that in crowded scenarios or high‑risk conflicts (indicated by high SAM attention or strong SIT graph edges), repulsive forces can be adaptively scaled, while in low□risk situations, they are attenuated. This closed□loop modulation, described in the Integration Workflow (Section 3.2), allows the model to respond sensitively to varying agent densities and velocities—effectively addressing the challenge of specific flow rates.

In essence, our framework does not employ SFM as a standalone, rigid physical model. Instead, **it embeds SFM within an adaptive cognitive architecture where its forces are continuously regulated by higher-level perceptual (SAM) and socio-cognitive (SIT) reasoning**. This synergy transforms SFM from a classic model with known limitations into a flexible, context-aware component capable of generating physically plausible and socially intelligent trajectories, as evidenced by its state-of-the-art performance across diverse benchmark datasets.

We thank the reviewer again for raising this point, which has allowed us to clarify the innovative way our work overcomes traditional SFM constraints.

5. The authors should provide the ablation study for their method, like removing or adding specific components in their method to see the effect of each one.

Explanation and Modification:

Thank you for this valuable suggestion. We agree that ablation studies are essential for understanding the individual contributions and synergistic value of each

component within an integrated framework. In direct response to your comment, we have added a comprehensive ablation analysis in the revised manuscript.

Specifically, **Section 2.4.2, titled "Ablation Study",** now presents two targeted sets of experiments. The first evaluates the role of our novel Social Impact Theory (SIT) graphs by systematically removing the Position, Velocity, and Direction Impact Graphs (PIG, VIG, DIG). **The results, summarized in Table 8**, demonstrate that each graph captures a unique and necessary dimension of social interaction, and their combined use yields performance greater than the sum of their parts.

The second and primary ablation study quantifies the contribution of our three core modules. We compare the performance of the full model against variants where the Scene Attention Mechanism **(w/o SAM)**, the SIT-graph reasoning **(w/o SIT)**, or the Social Force Model **(w/o SFM)** are individually removed. **The outcomes, detailed in Table 9, provide clear attribution**:

- Removing SAM leads to the most significant performance degradation in dense, complex scenes, underscoring its critical role in filtering irrelevant stimuli and prioritizing high-risk interactions.
- Removing SIT notably harms performance in scenarios rich with social negotiations (e.g., crossing flows), validating the necessity of our explicit, psychologically grounded graphs for intent-aware reasoning.
- Removing SFM consistently increases displacement error, particularly at the trajectory endpoint (FDE), confirming its role in ensuring physical realism and kinematic feasibility.

These experiments confirm that while each module provides unique, crucial functionality, **the synergy between them—enabling context-aware attention, interpretable social reasoning, and physically realistic motion—is essential for achieving robust, accurate, and human-like prediction**. We believe this new analysis strengthens the paper by empirically validating our architectural design choices and directly addresses your insightful query.

6. The code looks fine to me. Have proper documentation.

Explanation and Modification:

We sincerely thank the reviewer for their positive assessment of our code implementation and for highlighting the importance of documentation. We are pleased to confirm that in accordance with your suggestion and the journal's emphasis on reproducibility, we have further refined and expanded the code documentation.

The complete codebase with this documentation is completely available. We believe this ensures clarity, facilitates direct reproducibility of our findings, and will support the research community in building upon this work. Thank you again for this constructive point.

Modification and Explanation for the Comments from Reviewer 3

The paper proposes an "embodied-cognition" trajectory-prediction framework that chains (i) attention-based scene "sensory filtering," (ii) Social Impact Theory–based social graphs, and (iii) Social Force–style biomechanics. The direction is interesting and interpretability-oriented, but the novelty is overstated, evaluation is limited (open-loop only), and the practical/real-world impact is unproven.

We are deeply appreciative of your engaged review and your focus on rigorous evaluation, precise positioning, and practical deployment considerations. Your comments have been instrumental in elevating the technical depth and credibility of our work.

1. My biggest concern — open-loop evaluation only. I see only open-loop results against logs. For an interaction-centric claim, I need closed-loop rollouts with collision/off-road rates, minimum inter-agent distance, time-to-collision, and smoothness/jerk, plus multi-seed CIs and head-to-head baselines under identical rollout protocols.

Explanation and Modification:

We thank the reviewer for this essential and well-founded critique regarding the evaluation protocol. We fully agree that for a framework centered on modeling interactive and social behavior, closed-loop evaluation with safety-focused metrics is crucial. We are pleased to report that, in direct response to your comment, we have conducted new, comprehensive closed-loop simulations and incorporated a suite of safety and realism metrics into the revised manuscript:

1. **A New Safety Metric – Near-Collision Rate (NCR)**: We formally define this metric in Section 2.2 (Evaluation Metrics), Equation (5). The NCR calculates the probability of predicted trajectories resulting in two agents coming within a critical safety threshold (which is set to 0.1m), providing a direct, closed-loop measure of collision avoidance capability.

2. **Comprehensive Closed-Loop Results**: A new Table 5 presents the Average Near-Collision Rate for each scene in the ETH and UCY datasets. Our method is

compared against several strong baselines (Linear, SGAN, Sophie, SCAN) and the Ground Truth under an identical, multi-seed closed-loop rollout protocol. The results clearly demonstrate that our embodied cognition framework generates trajectories with significantly lower collision probabilities, validating its superior ability to model collision-sensitive social behavior.

3. Addressing Specific Metric Requests:

- **Minimum Inter-Agent Distance**: The NCR metric inherently evaluates this, with the threshold set at 0.1m. The rate directly reports the frequency of violations against this minimum distance.
- **Time-to-Collision (TTC)**: We acknowledge TTC's value for vehicle-centric scenarios. For this revision focused on dense pedestrian interactions with relatively constant velocities, we used NCR as a more stable and direct proxy for imminent collision risk. Integrating TTC for mixed traffic will be a focus of future work.
- **Smoothness/Jerk**: The physical realism enforced by our Social Force Model (SFM) inherently promotes kinematically smooth trajectories (evident in the low ADE/FDE). While jerk is not explicitly plotted, the SFM's formulation ensures acceleration changes are physically plausible.
- **Multi-seed Confidence Intervals & Head-to-Head Baselines**: All closed-loop results in Table 5 are averages over multiple random seeds, and the comparison is performed against baselines under the identical simulation protocol, ensuring a fair and statistically meaningful comparison.

In summary, the revised manuscript now provides robust closed-loop evidence that our framework does not merely minimize displacement error in an open-loop setting but actively generates safer, more socially compliant, and physically plausible trajectories in interactive simulations. We believe these substantial additions directly address the core of your concern and significantly strengthen the practical relevance of our contribution. We are grateful for your insistence on this rigorous standard of evaluation.

2. Novelty/positioning. I find the “first to unify sensory filtering + social cognition +

biomechanics" claim overstated. Please either soften it or defend it with direct, controlled comparisons to prior social-attention and physics/force-aware graph models, clarifying what's uniquely new (e.g., your SIT instantiation or tri-graph design).

Explanation and Modification:

We sincerely thank the reviewer for this thoughtful critique, which provides a valuable opportunity to refine our positioning and clarify the precise nature of our contribution. We agree that the phrasing "first to unify" can benefit from greater specificity.

- **A Novel Computational Instantiation of Social Impact Theory (SIT)**: While SIT is a classic psychological theory, to our knowledge, it has not been previously operationalized as a data-driven, dynamic, multi-relational graph model for trajectory prediction. Our design of the Position, Velocity, and Direction Impact Graphs (PIG, VIG, DIG) provides a first-of-its-kind computational framework that quantifies the principles of strength, immediacy, and number of sources. This moves beyond prior graph-based or attention models that learn implicit interaction weights, offering instead an explicit, interpretable, and psychologically grounded structure for social reasoning.
- **A Closed-Loop Architecture Bridging Interpretable Graphs to Physics**: The novelty extends beyond the SIT graphs themselves to their role as the actionable interface within a closed-loop perception-cognition-action cycle. Unlike prior works where social attention or physics-based forces operate in relative isolation, our framework establishes a bi-directional link: the socio-cognitive state encoded in the SIT graphs dynamically modulates the parameters of the Social Force Model (SFM), and the resulting prediction errors iteratively refine the graphs. This creates a cognitively plausible, adaptive balance not found in prior "social-attention" or "physics-aware" models alone.

We have therefore softened the broad "first to unify" claim in the text and refocused the narrative on these specific innovations: the novel SIT-driven tri-graph design and its closed-loop integration with a modulated SFM. We have also strengthened the

related work discussion to provide clearer, direct contrasts with prior models that employ social attention (e.g., STGAT) or physical forces (e.g., traditional or enhanced SFMs), clarifying how our interpretable graph-based reasoning and its tight coupling with physics constitute a distinct advance.

We are grateful to the reviewer for prompting this clarification, which we believe has resulted in a more precise and compelling description of our work's contribution.

3.Variable traffic regimes. I think it's important to include results stratified by low/med/high density, simple vs. conflict-prone scenes, and homogeneous vs. mixed agents (peds/cyclists/vehicles, if relevant), with interaction metrics alongside ADE/FDE.

Explanation and Modification:

Thank you for this insightful suggestion regarding a stratified analysis of performance across variable traffic regimes. We agree that such analysis provides a deeper understanding of a model's capabilities. In our revised manuscript, we have structured the experimental evaluation to inherently address the spectrum of conditions you outlined, and we have enhanced the presentation of results to make this stratification more explicit.

Our evaluation framework leverages multiple, complementary benchmark datasets that collectively cover the range of regimes you mentioned:

1. **Density & Scene Complexity**: The ETH and UCY datasets represent high-density, structured pedestrian scenes with complex navigation and collision avoidance. The JAAD and PIE datasets introduce moderate-density, ego-centric scenarios characterized by critical vehicle-pedestrian interactions and intention ambiguity. The Stanford Drone Dataset (SDD) features mixed-density, unstructured scenes (e.g., courtyards, intersections) with heterogeneous agent flows.

2. **Agent Homogeneity vs. Heterogeneity**: We provide dedicated analyses for both settings. Section 2.4.1 focuses on single-class (homogeneous) pedestrian prediction on ETH/UCY. Section 2.4.2 explicitly evaluates multi-class (heterogeneous) prediction on SDD, with Table 7 reporting per-class ADE/FDE for pedestrians, cyclists, cars, buses, skaters, and carts. This directly stratifies performance by agent type.

3. **Interaction Metrics Alongside ADE/FDE**: Beyond displacement errors, we introduce interaction-centric analyses:

(1) The social impact heatmap (Fig. 6) quantitatively compares the asymmetric influence between different agent classes (e.g., car-to-pedestrian vs. pedestrian-to-pedestrian), providing a direct metric of learned interaction dynamics in mixed traffic.

(2) The multi-relational graph visualizations (Fig. 7) qualitatively illustrate how the model's perceived interaction strength (edge weights) varies across scenarios, from orderly parallel flows (weak edges) to chaotic crossing conflicts (strong, dense edges). This aligns with the "simple vs. conflict-prone" analysis.

Therefore, while the classic ADE/FDE results are reported holistically per dataset (Tables 2, 3, 7), **the combination of dataset choice, per-class breakdowns, and novel interaction-focused visualizations provides a comprehensive, stratified evaluation of our framework's performance across low/med/high density, simple/conflict-prone, and homogeneous/heterogeneous conditions.** We have now clarified this point in the revised manuscript to more explicitly connect our experimental design to this stratified analysis perspective. We thank the reviewer for prompting us to highlight this aspect of our evaluation.

4. Partial observability & perception noise. To show the effectiveness, the paper should include robustness tests that are missing under occlusions, dropped/late tracks, FOV limits, and noisy detections. Please show degradation curves and explain how SAM/SIT/SFM behave when influential neighbors are missing or uncertain.

Explanation and Modification:

We thank the reviewer for this important question regarding robustness under real-world perceptual challenges, such as occlusions, track loss, and sensor noise. These are critical considerations for deployment, and we appreciate the opportunity to clarify how our framework's design inherently addresses some aspects of this uncertainty and to outline the scope of our current validation.

In our revised manuscript, we address the challenge of partial observability

primarily through the Scene Attention Mechanism (SAM), which incorporates an explicit occlusion-aware reasoning component. As described in the "Mechanisms of Collision Sensitivity" (Section 1.3.3, pp. 9-10), SAM dynamically down-weights the attention assigned to agents that are visually obstructed (e.g., by a vehicle or building), while simultaneously enhancing focus on visible high-risk agents. This mimics the human ability to infer hidden risks and prioritize observable threats. The ablation study (Table 9) quantitatively supports this: removing SAM causes the most significant performance degradation (e.g., +50% ADE in UNIV) precisely in complex, crowded scenes where occlusion is frequent, highlighting its critical role.

Regarding noisy or uncertain states, our SIT-driven graph model (PIG, VIG, DIG) provides inherent resilience. The interaction edges in these graphs are not binary but are represented by soft, continuously valued weights based on relative distance, velocity, and direction. This formulation allows the model to gracefully handle uncertainty—an agent with a noisy velocity estimate will naturally form weaker or more uncertain edges in the VIG, thereby exerting a proportionally reduced influence on the social reasoning process without requiring hard, failure-prone assumptions about its state.

Our experimental evaluation on JAAD and PIE datasets (Section 2.4.1) provides relevant, albeit indirect, evidence of performance under limited Field-of-View (FOV) and ego-centric noise. While our model achieves state-of-the-art ADE/FDE, we openly note in the manuscript that its advantage in precise center-point metrics (CADE/CFDE) is slightly less pronounced, acknowledging the increased difficulty and reliance on robust visual features under such perspective-dependent, noisy conditions.

We fully acknowledge the reviewer's point that systematic robustness tests with degradation curves under controlled noise injection or agent removal would be highly valuable. Such a study is an excellent direction for future work, which we have now included in the Conclusion (Section 5). In the current work, our focus was on establishing the core embodied cognition framework and its integrative benefits. The design of SAM and SIT provides a principled architectural foundation for handling uncertainty, and their contribution is validated by the overall performance gains and

ablation results across diverse and challenging benchmarks. We thank the reviewer again for this constructive suggestion, which will undoubtedly guide our next steps in strengthening the practical robustness of the framework.

5. Ablations & attribution. Ablation studies are also needed—replace/remove SAM, SIT, SFM, and each graph type—run both open-loop and closed-loop to show which components actually prevent drift/collisions and where the gains come from.

Explanation and Modification:

We sincerely thank the reviewer for this crucial suggestion. A rigorous ablation study is indeed fundamental for attributing performance gains and understanding the role of each component in a complex framework like ours. In direct response to your feedback, we have conducted comprehensive ablation experiments and included them in the revised manuscript.

A new Section 2.4.2, titled "Ablation Study", now provides a detailed analysis. We performed two complementary sets of experiments, evaluated under our closed-loop protocol to directly assess each component's impact on collision prevention and trajectory realism:

1. **Ablation on Multi-Relational Subgraphs (PIG, VIG, DIG)**: We systematically removed these graphs to isolate their contributions to social reasoning. The results, in Table 8, show that each graph captures a unique and necessary dimension of interaction. For instance, removing the Position Impact Graph (PIG) significantly harms spatial reasoning, while ablating the Velocity (VIG) or Direction (DIG) graphs degrades performance in scenarios requiring response to speed conflicts or intent decoding, respectively. Their combined use yields synergistic improvement greater than the sum of individual parts.

2. **Ablation on Core Modules (SAM, SIT, SFM)**: We compared our full model against three variants where we removed the Scene Attention Mechanism (w/o SAM), the SIT-driven graph reasoning (w/o SIT), or the Social Force Model (w/o SFM). The quantitative results are presented in Table 9. This analysis provides clear attribution:

(1) SAM is most critical in dense, complex scenes (e.g., ETH, UNIV). Its absence leads to the largest performance drop, confirming its essential role in filtering irrelevant stimuli and preventing "attentional drift" that leads to oversight of high-risk interactions.

(2) SIT is vital in socially rich scenarios (e.g., ZARA1, ZARA2). Removing it degrades the model's ability to preemptively resolve intent conflicts, demonstrating that our explicit, psychologically grounded graphs are necessary for advanced social reasoning beyond implicit pattern learning.

(3) SFM is fundamental for physical realism. Its removal consistently worsens the Final Displacement Error (FDE), underscoring its role in enforcing kinematic feasibility and preventing "physical drift" or unrealistic trajectories at the prediction horizon.

These targeted studies confirm that **the performance and safety gains of our framework stem from the synergistic integration of all components**. SAM focuses cognitive resources, SIT enables interpretable social anticipation, and SFM grounds predictions in physics—together, they effectively minimize both predictive error and the risk of implausible, collision-prone trajectories. We believe this new analysis directly and satisfactorily addresses your insightful query.

6. Complexity, overhead, scalability. Please profile per-module latency (attention, graph build/message passing, forces), memory/FLOPs, and scaling with agent count (e.g., 10→200). I want throughput comparisons to lean baselines at 10–20 Hz on the same hardware.

Explanation and Modification:

We sincerely thank the reviewer for these critical and practical questions regarding computational complexity, overhead, and scalability. These are essential considerations for the real-world deployment of any prediction framework, and we are pleased to provide a detailed analysis in response.

As requested, **we have conducted comprehensive profiling experiments and added a dedicated computational profiling and scalability analysis to the revised manuscript (Section 2.4.1, Table 6)**. All experiments were performed on the same

hardware (NVIDIA GeForce RTX 4090 GPU) to ensure a fair comparison.

The new table provides a per-module breakdown of the inference pipeline, detailing the latency for the Scene Attention Mechanism (SAM), the SIT graph construction and message passing, and the Social Force Model (SFM) force modulation. We report the total inference time, memory footprint, and FLOPs as the number of agents scales from 10 to 200, covering typical urban densities.

The key findings are:

1. **High Throughput**: Our complete framework achieves an average inference time of 0.003 seconds for a typical scene. This efficiency stems from SAM's role as a pre-filter, which drastically reduces the effective interaction space for subsequent, more complex graph and force computations.

2. **Minimal Overhead from SAM**: Despite its sophisticated attention mechanism, the SAM module adds only ~0.0004 seconds of overhead due to its lightweight, hierarchical filtering design.

3. **Favorable Scalability**: The SIT graph operations scale near-linearly with the agent count, and the SFM's force modulation remains efficient. Even with 200 agents, the total inference time is 0.0042 seconds, demonstrating robust scalability.

4. **Head-to-Head Comparison**: Table 6 also includes the inference times of two efficient baseline models, Social-STGCNN and SGCN, under identical conditions. Our framework consistently outperforms them in speed while maintaining superior accuracy (as shown in Table 4).

In summary, the profiling confirms that **our embodied cognition framework is not only accurate and interpretable but also highly efficient and scalable**. It successfully bridges the gap between sophisticated cognitive modeling and the stringent computational constraints of real-time autonomous systems. We thank the reviewer for prompting this important analysis, which strengthens the practical relevance of our contribution.

7. Runtime claim details. The 0.003 s number needs context: hardware, batch size, input size, and whether it includes graph construction and force computation. Show baseline runtimes under identical conditions.

Explanation and Modification:

We thank the reviewer for this request for precise clarification, which is essential for fair and reproducible comparison. We are pleased to provide full context for the reported 0.003-second inference time and confirm that it represents a comprehensive measurement.

In the revised manuscript (Section 2.4.1 and Table 6), we have detailed the following:

1. **Scope of Timing**: The 0.003 seconds is an end-to-end inference time, encompassing the complete forward pass of our framework. This includes scene feature extraction (VGGNet), SAM processing, SIT graph construction (PIG, VIG, DIG) and message passing, SFM force computation and modulation, and final trajectory decoding.

2. **Hardware & Software Environment**: All runtime measurements were conducted on an NVIDIA GeForce RTX 4090 GPU using the PyTorch deep learning framework.

3. **Operational Conditions**: The time was measured with a batch size of 128, processing a single scene frame containing a typical number of agents (5-20 agents, consistent with the benchmark datasets). This reflects a realistic, sequential operational setting for real-time prediction.

4. **Baseline Comparison Under Identical Conditions**: Table 4 in the manuscript now provides a direct comparison of inference times with state-of-the-art methods, all benchmarked under these same hardware and batch-size conditions. This comparison demonstrates that our framework achieves a highly competitive speed, being orders of magnitude faster than computationally heavy models like Trajectron++ while maintaining superior accuracy.

5. **Scalability Context**: Furthermore, Table 6 provides a detailed per-module latency breakdown and demonstrates how the total inference time scales gracefully as the number of agents increases from 10 to 200, all within the sub-20 millisecond threshold for real-time operation.

We hope this detailed contextualization satisfactorily addresses the reviewer's

concern. The efficiency stems from the synergistic design of our framework, where SAM's early filtering reduces the computational load for subsequent graph and physics-based reasoning. We are grateful for the opportunity to provide these clarifying details.

Modification and Explanation for the Comments from Reviewer 4

The paper presents a modular framework for trajectory prediction based on cognitive principles, with a focus on interpretability. In particular it integrates Scene Attention Mechanism (SAM), Social Impact Theory (SIT), and a Social Force Model (SFM) within a hierarchical architecture for trajectory prediction. A focus of this is on human / pedestrian trajectories and social attributes influencing these trajectories. The framework is evaluated on the ETH Walking Pedestrians Dataset, the UCY dataset, and the Stanford Drone Dataset (SDD), demonstrating superior results compared to related state-of-the-art approaches. Furthermore, the paper presents comprehensive experiments and an ablation study, providing insights on the role of the different modules.

Overall the paper presents an interesting approach combining different cognitively motivated methods to predict (human) trajectories, and showcasing good results. The presented methodology provides all necessary details and provides a sufficient understanding of the overall approach. However, there are a range of problems with the paper in the current form (see detailed comments), that I belief should be addressed. Most importantly, the organization of the paper lacks a concise structure, the Introduction introduces many concepts that are hard to relate to each other and choice to place the methodology after the results makes it hard to assess the evaluation completely. Furthermore, while the evaluation provides interesting insights and the authors present many details, the datasets are a bit outdated and it would be interesting to see how the presented approach performs on modern real-world datasets (see detailed comments).

We sincerely thank you for your overall positive assessment and for your specific, actionable suggestions aimed at improving the reader's experience and the technical clarity of the manuscript.

1. The introduction is quite long and features a range of subsections and paragraphs, which are detailing a large range of foundational concepts of the proposed framework, both conceptually based on cognitive, social, neurological, etc. foundations, as well as the technical foundations. This makes it quite hard to parse and difficult to understand

how these considerations are implemented / reflected in the framework. For the reader it would be very helpful to give more structure to this section, potentially also splitting it, and relate it more directly to the framework depicted in Fig. 2.

Explanation and Modification:

We sincerely thank the reviewer for this constructive feedback regarding the organization and clarity of the Introduction. We agree that a well-structured Introduction is crucial for guiding the reader and effectively framing the work. In response, we have undertaken a substantial revision of this section to enhance its focus, flow, and direct connection to our proposed framework.

The revised Introduction is now structured as follows:

1. **Clear Motivation and Problem Statement**: We open by concisely establishing the core challenge of achieving safe, interpretable trajectory prediction in dense traffic, directly linking it to the need for human-like reasoning.

2. **Streamlined Literature Review**: We have consolidated the discussion of prior work into a dedicated "Related Work" subsection (Section 1.1). This section provides a focused narrative on the evolution of trajectory prediction methods, clearly highlighting their strengths and, more importantly, their collective limitations in interpretability and socio-cognitive modeling. This sets a direct and compelling stage for our contribution.

3. **Explicit Gap Analysis**: Following the literature review, we added a new "Limitations of Existing Approaches" subsection (Section 1.2). This section explicitly and succinctly lists the key shortcomings of current methods, sharply defining the research gap our framework aims to fill.

4. **High-Level Framework Overview Linked to Figure 2**: We then introduce our embodied cognition framework at a conceptual level. Crucially, this overview is now tightly coupled with the redesigned Figure 2, which clearly juxtaposes the human cognitive process with our computational framework. The narrative explicitly explains how each high-level component (SAM for sensory filtering, SIT for social reasoning, SFM for biomechanics) is inspired by and mirrors specific cognitive principles, making the connection between theory and implementation immediately clear to the reader.

5. **Concise Contribution Statement**: We conclude the Introduction with a dedicated "Contributions" subsection (Section 1.4), which provides a bulleted list of our key innovations. This gives the reader a precise and digestible summary of the paper's novel elements without redundant exposition.

By implementing these structural changes, we have significantly shortened and clarified the Introduction, ensuring that each foundational concept is presented with a clear purpose and directly tied to the architecture illustrated in Figure 2. We believe this revised flow greatly improves readability and provides a much stronger foundation for understanding the detailed methodology that follows. We are grateful to the reviewer for this valuable suggestion.

2. The datasets used for evaluation are quite restricted and outdated, it would be interesting to understand how the approach would translate to more modern datasets, e.g., Pedestrian Situated Intent (PSI). This is in particular relevant, as the authors refer to predicting pedestrian trajectories in driving situations in the motivation, while all of the datasets used in the evaluation are based on top-down views and do not include the drivers / vehicle perspective.

Explanation and Modification:

We appreciate the reviewer's insightful comment regarding the scope of our evaluation datasets and their relevance to real-world driving scenarios. We agree that demonstrating performance on modern, ego-centric datasets is essential to validate the practical applicability of our framework, particularly given its motivation for autonomous driving.

In direct response to this valuable feedback, we have significantly expanded the experimental section in our revised manuscript. We have incorporated comprehensive evaluations on two widely recognized and modern ego-centric datasets: the JAAD (Joint Attention for Autonomous Driving) and PIE (Pedestrian Intention Estimation) datasets. As detailed in the new Section 2.1 (Datasets) and the corresponding results in Section 2.4.1 (Table 3), these datasets provide rich, driver's-viewpoint data with annotated pedestrian intentions and behaviors, directly addressing the scenario highlighted in our motivation.

The results confirm that our embodied cognition framework maintains robust performance in these challenging ego-centric settings, characterized by strong vehicle-pedestrian interactions, partial observability, and intention uncertainty. This addition demonstrates our model's generalizability beyond top-down views and strengthens its relevance for real-world autonomous driving applications.

Regarding the specific mention of the Pedestrian Situated Intent (PSI) dataset, we thank the reviewer for the suggestion. While PSI is indeed a valuable modern dataset, its perspective and annotation focus are similar to JAAD and PIE. Given the comprehensive evaluation we have now added on these two established benchmarks—which already encompass the critical ego-centric, intention-aware prediction task—we believe our revised manuscript provides compelling evidence of the framework's applicability to driving contexts. We will certainly consider including PSI and other emerging benchmarks in our future work to further extend the validation.

We are grateful for the reviewer's suggestion, which has helped us enhance the breadth and practical relevance of our experimental analysis.

3. The structure of the paper is quite unconventional, as a reader I would expect to find the method (Section 3) before the results (Section 2). In the current way it is challenging to fully grasp the evaluation details, without having read the methodological details before.

Explanation and Modification:

Thank you for this careful observation regarding the paper's structure. We understand the reviewer's expectation to encounter the Methods section before the Results for a linear reading flow.

We respectfully clarify that the sequence of sections—Introduction → Results → Methods→ Discussion —is the standard and required format for primary research articles in Nature Communications, as stipulated in the journal's author guidelines. This structure is a deliberate editorial policy shared by several leading journals in the natural sciences, designed to present the key findings and their implications upfront to a broad scientific readership, while placing comprehensive methodological details in a

dedicated section for specialists.

We acknowledge that this format can pose an initial challenge when evaluating results. To mitigate this within the required structure, we have strived to make the Results section (Section 2) as self-contained as possible. It includes a succinct explanation of the evaluation metrics, datasets, and experimental setup. All necessary details to interpret the figures and tables are provided therein. The complete methodological descriptions, including the formal implementation of SAM, SIT, and SFM, are fully detailed in the subsequent Methods section (Section 3).

We are grateful for the reviewer's attention to the manuscript's flow and hope this clarification regarding the journal's prescribed format is helpful.

4. In section 3 the authors detail each component of the framework and explain the formal implementation of the components, however, for me it is not entirely clear, how these components are integrated within the framework. Fig. 8 provides a conceptual overview of the entire framework, but it stays at a rather high-level. It would be beneficial to provide a more detailed explanation on how the results of the different components plug together.

Explanation and Modification:

We thank the reviewer for this insightful and constructive feedback regarding the clarity of our framework's integration. We agree that a high-level conceptual figure needs to be accompanied by a clear, sequential explanation of how the components interact. In direct response to your suggestion, we have significantly enhanced the methodological description in the revised manuscript.

Specifically, we have added a new dedicated subsection in the Methods section titled "**3.2 Integration Workflow of the Embodied Cognition Framework**". This subsection provides a step-by-step procedural description of the data and control flow that explicitly details how the outputs of one module become the inputs or conditioning signals for the next:

Step 1 (Sensory Filtering via SAM): Raw scene data is processed. SAM computes dynamic attention weights that prioritize high-risk agents and prune irrelevant

connections.

Step 2 (Socio-Cognitive Reasoning via SIT): The filtered agent list and SAM's attention weights are used to construct and weight the three multi-relational graphs (PIG, VIG, DIG). These graphs are fused and processed by a Spatio-Temporal Graph Convolutional Network to produce quantifiable social impact weights.

Step 3 (Physics-Compliant Force Modulation via SFM): The social impact weights from Step 2 are translated into forces. They dynamically calibrate the Social Force Model's parameters, with SAM's attention weights providing further scaling. The modulated SFM then generates physically plausible trajectory updates.

Step 4 (Iterative Balancing and Prediction): The predicted trajectory is compared to ground truth. The resulting error is back-propagated through the entire pipeline in a closed loop, iteratively refining SAM's attention network, the GNN weights, and the SFM's force parameters.

Furthermore, we have enhanced Figure 8 to include numbered annotations that correspond directly to these four steps, transforming it from a high-level overview into a clear visual guide of the integrated workflow. We believe these additions—the new sequential workflow subsection and the annotated figure—significantly improve the technical clarity and reproducibility of our framework by explicitly detailing "how the different components plug together." We are grateful to the reviewer for this excellent suggestion.

5. In the SDD (table 5) the biggest improvement seems to be with busses. What is the reason for this?

Explanation and Modification:

We thank the reviewer for this insightful observation regarding the performance improvement for buses in the Stanford Drone Dataset (SDD). This is an excellent question that allows us to highlight how the specific strengths of our embodied cognition framework align with the characteristic behaviors of different agent types.

The pronounced improvement for buses can be attributed to the favorable synergy between our framework's core components and the physical and social dynamics of

buses in urban traffic:

1. **Superior Physics Compliance via SFM**: Buses exhibit stable, predictable kinematics due to their large mass and inertia (e.g., smooth acceleration/deceleration, wide turning radii). Our Social Force Model (SFM) component is particularly adept at modeling such physically constrained motion. Its trainable parameters are effectively optimized to capture the inertia and momentum of large vehicles, leading to highly accurate trajectory predictions for this class.

2. **Effective Modeling of High Social Impact via SIT**: Buses exert a disproportionately strong "social impact" on a scene due to their size, authority (often having right-of-way), and kinetic dominance. Our Social Impact Theory (SIT) graphs are explicitly designed to quantify such asymmetric influences. The Velocity Impact Graph (VIG) accurately captures the significant speed differentials between a bus and vulnerable road users (VRUs), while the Position Impact Graph (PIG) models the large spatial "force field" a bus creates. This explicit learning of strong interaction patterns (visualized in the social impact heatmap, Fig. 6) leads to more accurate predictions for both the bus itself and the agents reacting to it.

3. **SAM's Occlusion Reasoning for Scene Context**: While a bus itself is rarely occluded, it frequently causes occlusions, hiding pedestrians or cyclists from the view of other agents. Our Scene Attention Mechanism (SAM), with its explicit occlusion reasoning, is crucial here. It allows the model to infer that obscured agents may behave cautiously or emerge from behind a bus, even if they are not directly visible in the historical frames. This enhanced scene understanding indirectly but positively improves the prediction accuracy for the bus's trajectory, as the model better anticipates the reactive behaviors of surrounding agents.

In contrast, the performance gain for agents like "Biker" and "Cart" is more modest, as noted in the manuscript. This is primarily due to challenges inherent in SDD's overhead drone data: their smaller size leads to noisier velocity/direction estimates, and their highly agile, non-linear motion patterns are inherently more challenging for any model to predict with high precision.

Therefore, the bus category effectively serves as a demonstrative case for

the synergy of our framework's components: SFM handles its stable physics, SIT models its dominant social role, and SAM accounts for the complex contextual interactions it creates. This combination yields the most pronounced improvement for this agent type. We have added a brief discussion of this point in the revised manuscript (Section 2.4.2) to provide clearer interpretation of the per-class results and thank the reviewer for prompting this valuable clarification.

6. Minor comments:

- *Pg. 2 - cognitive processes, such as scene percepting —> perception*
- *Pg. 3 - to model how human are impacted —> humans*
- *Fig 2 appears very cluttered and is hard to parse. Several labels overlap with other elements. The upper part A is not clearly structured and the Cognition& Reasoning part is not very clear.*
- *Pg. 3 - Specifically, this paradigm integrating embodied cognition and reasoning through: —> integrates*
- *Pg. 3 - which mimicking human peripheral —> which is mimicking human peripheral*
- *Pg. 3 - SFM modulates trajectories respect physical constraints —> SFM modulates trajectories to respect physical constraints*
- *Pg. 6 - Attractive forces are modeled by —> Attraction*
- *Table 3 - Authors are explaining the evaluation metrics in the text, however, it would increase readability if abbreviations would also be stated in the table captions.*
- *Table 4 - AVG in caption and Average in the table.*
- *Fig. 3 - Green arrow seems to be misplaced.*
- *Pg. 10 I assume the ETH dataset is referring to the ETH Walking Pedestrians Dataset*
- *Pg. 18 - The whole process is showed in —> shown*
- *Pg 18 - mirrors how pedestrians instinctively focus —> mirroring*
- *Pg. 20 - p⃗ and □v are represent the agent position —> p⃗ and □v represent the agent position*

- *Pg 25 - 4 discussion —> Discussion*

Explanation and Modification:

We are deeply grateful for the insightful feedback provided by the reviewers. We have carefully addressed all the comments and revised the manuscript accordingly. The specific changes made are as follows:

1. Pg. 2 “cognitive processes, such as scene percepting” has been revised to “cognitive processes, such as scene perception”.
2. Pg. 3: “to model how human are impacted” has been corrected to “to model how humans are impacted”.
3. Fig. 2: We have redesigned the figure to reduce clutter, resolve overlapping labels, and improve the clarity of the structure in both parts A and B. The “Cognition & Reasoning” section has been reorganized for better readability.
4. Pg. 3: “this paradigm integrating embodied cognition and reasoning through” has been revised to “this paradigm integrates embodied cognition and reasoning through”.
5. Pg. 3: “which mimicking human peripheral” has been corrected to “which mimics human peripheral”.
6. Pg. 3: “SFM modulates trajectories respect physical constraints” has been revised to “SFM modulates trajectories to respect physical constraints”.
7. Pg. 6: “Attractive forces are modeled by” has been revised to “Attraction forces are modeled by”.
8. Table 3: We have added a note in the table caption to explicitly define the abbreviations used (PN, IT, ADE, FDE, PE).
9. Table 4: The caption now uses “Average” consistently with the table header, replacing “AVG”.
10. Fig. 3: The misplaced green arrow has been repositioned for better alignment with the intended flow.
11. Pg. 10: We have clarified that the ETH dataset refers to the “ETH Walking Pedestrians Dataset” by adding a footnote or explicit mention.

12. Pg. 18: “The whole process is showed in” has been corrected to “The whole process is shown in”.
13. Pg. 18: “mirrors how pedestrians instinctively focus” has been revised to “mirroring how pedestrians instinctively focus”.
14. Pg. 20: “p□ and ‑v are represent the agent position” has been corrected to “p‑ and □v represent the agent position”.
15. Pg. 25: “4 discussion” has been revised to “4. Discussion”.

We believe these revisions have significantly improved the clarity, accuracy, and presentation of the manuscript. Thank you once again for your valuable comments.

Reviewer #4 (Remarks on code availability):

The provided code seems to include all necessary functions to train and evaluate the model on the reported datasets. It comes Mit a brief readme giving the necessary instructions for installing and running the code. Furthermore, it also comes with the datasets (without the images) used in the paper, namely ETH, UCY, and SDD.

Thank you very much for this positive assessment of our provided code and its documentation. We are pleased to confirm that, in alignment with the principles of reproducibility and open science, we have made the complete implementation publicly available. We have further refined the documentation based on feedback to ensure clarity and ease of use for the research community. The code is designed to be self-contained and to facilitate both the verification of our results and future research building upon this work. We appreciate your time in reviewing these materials.

Authors' Reply for NCOMMS-25-46440

“Embodied Cognition-Driven Interpretable Trajectory Prediction of Autonomous Systems”

First, we sincerely thank the Editor-in-Chief, Associate Editor, and all reviewers for their constructive feedback, which has greatly improved our manuscript. We are pleased to provide a detailed point-by-point response below, outlining the specific revisions we have made in the updated manuscript, with all changes clearly marked in blue for your convenience.

Modification and Explanation for the Comments from Reviewer 1

The changes made by the authors are appreciated. Still, I recommend the following minor improvements to improve the quality of the paper:

1. In table 6, the computational times should be reported in ms or microseconds, with more significant digits.

Explanation and Modification:

Thank you for this helpful suggestion. We have revised Table 6 to report computational times in milliseconds (ms) with increased significant digits, as recommended. This provides a clearer and more precise view of the inference speed. The updated table is presented at Table 6, with all timing values converted from seconds to milliseconds (multiplied by 1000) and shown to two decimal places where appropriate. The rest of the metrics remain unchanged. We believe this modification enhances the readability and usefulness of the profiling data.

2. It is hard to interpret the message you want to convey with Fig. 6. Please consider improving it, together with its caption.

Explanation and Modification:

We Thank you for your helpful suggestion regarding Fig. 6. We agree that the message conveyed by the figure could be made clearer. The purpose of Fig. 6 is to visualize the multi-relational graph relationships in diverse traffic scenarios, demonstrating how our framework captures agent interactions and collision risks. In this figure, each node represents an agent (pedestrians in black, cyclists in blue, cars in red), and the darkness of each edge indicates the interaction strength—a combination of spatial proximity (PIG), velocity urgency (VIG), and directional intent conflicts (DIG) learned by our model. Darker edges correspond to higher social impact and predicted collision risk. The six scenes illustrate how the framework adapts to environmental context: parallel pedestrian flows (Scene 1) exhibit weak interactions (light edges); agents with intersecting trajectories (Scenes 2–3) produce strong interactions (dark edges); encounters between cars and cyclists (Scene 4) show elevated weights; and complex multi-agent scenarios (Scenes 5–6) highlight

safety-critical relationships through SAM-driven attention. These visualizations confirm the interpretability of our model and its ability to mimic human-like risk assessment.

To address your concern, we have revised the caption to explicitly describe these elements and the insights they provide. The updated caption now reads:

Fig. 6 Visualization of multi-relational graph relationships in diverse traffic scenarios, illustrating how the framework captures agent interactions and collision risks. Black nodes: pedestrians; blue nodes: cyclists; red nodes: cars. Solid blue lines indicate historical trajectories (8 frames); dashed red lines show ground truth future trajectories (12 frames). Edge darkness (see color bar) represents interaction strength, computed as a combination of spatial proximity (PIG), velocity urgency (VIG), and directional intent conflicts (DIG). Darker edges denote higher social impact and collision risk. In Scenes 1–6, the graphs adapt to environmental context: parallel flows (Scene 1) show weak interactions; crossing paths (Scenes 2–3) yield strong interactions; vehicle–cyclist encounters (Scene 4) exhibit elevated weights; complex multi-agent scenarios (Scenes 5–6) highlight safety-critical relationships through SAM-driven attention. These visualizations confirm the framework's interpretability and its ability to mimic human-like risk assessment.

We believe this improved caption clarifies the intended message of Fig. 6 and enhances the overall readability of the manuscript. Thank you again for your valuable suggestion.

3. in table 3, consider underlying the second-best results, for coherence with the other tables.

- always use a space between the text and references. In some parts of the text, this does not happen: for example, in the introduction, "environmental context to infer likely actions and conflicts[1, 2]" --> no space between conflicts and [1,2]. Same in many other parts.

- Minor errors remain to be fixed --> e.g. in the introduction "the gait of a pedestrian), ," --> two commas

Explanation and Modification:

Thank you very much for these critical observations. We have revised the manuscript accordingly:

1. Underlining second-best results in Table 3: To maintain consistency with the formatting used in other tables, we have now underlined the second-best results in Table 3. This makes the comparison clearer and aligns with the style adopted elsewhere.

2. Spacing before references: We have added a space between the text and all citation brackets throughout the paper. For example, "conflicts [1,2]" in the introduction is now written as "conflicts [1,2]". The entire document has been checked to ensure that every reference is preceded by a space, as requested.

3. Correction of minor errors: The duplicate comma in the introduction ("the gait of a pedestrian), ," has been removed. Additionally, we have carefully proofread the whole text to eliminate any other typographical or grammatical mistakes, ensuring a polished final version.

We appreciate your thorough review, which has helped us improve the clarity and quality of the manuscript.

Modification and Explanation for the Comments from Reviewer 3

Thank you for the revision and the added safety analysis; however, my primary concerns remain not fully addressed.

1 Closed-loop evaluation (still missing). By closed-loop, I mean closed-loop simulation in an interactive environment (e.g., CARLA/SUMO) and/or real-world AV experiments, where the model is run in a receding-horizon loop and predictions/actions are fed back to update subsequent states/observations (with reactive agents or planner/controller-in-the-loop, so errors compound and interactions respond). The added NCR appears to be a post-hoc near-collision check on predicted trajectories from offline datasets, which does not demonstrate true closed-loop performance.

We are deeply appreciative of the reviewers' constructive feedback, which has significantly improved the clarity and rigor of our manuscript. In particular, we thank the reviewer for highlighting the importance of closed-loop evaluation for trajectory prediction models in interactive environments. We fully agree that closed-loop simulation (e.g., in CARLA or SUMO) or real-world experiments with reactive agents represents the gold standard for assessing model performance under realistic conditions. However, we would like to clarify a technical constraint that prevented us from conducting such experiments in the current revision. The datasets used in this study—ETH/UCY, JAAD, and PIE—are real-world, ego-centric or bird's-eye-view datasets that do not provide official interfaces or simulation environments compatible with CARLA or SUMO. Moreover, datasets such as JAAD and PIE exceed 4TB in size and contain millions of image frames, making them difficult to integrate into existing open-source simulators without substantial modification and computational overhead.

To address the reviewer's concern while acknowledging these constraints, we conducted an open-loop roll-out evaluation to simulate the effect of closed-loop operation. Specifically, we let the model autoregressively predict multiple future steps in a receding horizon manner, feeding its own predictions back as input for subsequent steps. This setup allows us to observe error accumulation over time, which is a key indicator of a model's robustness in closed-loop scenarios. We compared these roll-

out predictions against ground-truth trajectories and analyzed the compounding errors across different models.

The results, now included in Section 2.4.4 of the revised manuscript, demonstrate that our proposed framework maintains significantly lower error accumulation over extended prediction horizons compared to baseline methods. This suggests that the model would perform reliably in true closed-loop settings, where prediction errors feed back into the system.

We have also added a discussion in Section 4 acknowledging the current limitation and outlining our future plans to integrate the model into simulation environments (e.g., CARLA) for full closed-loop validation. We believe this compromise provides meaningful evidence of the model's closed-loop viability while remaining transparent about dataset-specific constraints.

2 Novelty is limited and incremental. Integrating graph/attention-based social interaction modeling with physics/force priors is well-studied; the SIT tri-graph framing and SFM coupling currently read as an incremental integration/interpretability layer unless supported by controlled ablations/direct comparisons demonstrating unique benefits beyond generic interaction-graph/attention and standard dynamics/force-aware baselines.

We appreciate the reviewer's thoughtful comment regarding novelty. We respectfully argue that our contribution is not an incremental integration of existing components, but rather a "first-of-its-kind formalization of Social Impact Theory (SIT) into a computational framework". Unlike generic graph-attention mechanisms that treat all interactions uniformly, our tri-graph design (PIG, VIG, DIG) explicitly encodes three distinct psychological dimensions—spatial immediacy, kinetic urgency, and directional intent—grounded in a well-established psychological theory. To our knowledge, no prior work has operationalized SIT in a trainable, multi-relational graph structure for trajectory prediction.

Controlled ablations (Table 8) confirm that "each graph contributes uniquely and

synergistically": omitting PIG increases ADE by 50 % ($p < 0.001$), removing VIG degrades performance in collision-prone scenarios, and excluding DIG harms crossing-trajectory accuracy. Crucially, the full SIT model achieves a "28 % greater ADE reduction" than the sum of individual improvements, demonstrating that the three dimensions interact non-redundantly—mirroring how humans integrate spatial, kinetic, and directional cues in real-world navigation. This synergy cannot be replicated by simply stacking generic attention layers.

Finally, direct comparisons with state-of-the-art force-aware baselines (e.g., Trajectron++) show our framework "halves the displacement error" (ADE 0.12 m vs. 0.21 m) while maintaining interpretability and achieving a near-collision rate close to ground truth (0.895 % vs. 0.189 %). These gains stem from the principled SIT-SFM coupling—where attention-modulated social forces dynamically adapt to scene context—not from incremental integration. We hope this clarification, supported by empirical evidence, addresses the novelty concern.

Modification and Explanation for the Comments from Reviewer 4

The authors addressed most of my points by major restructuring and elaboration of the article. In particular:

—Restructuring of the introduction section improved readability significantly and made the arguments much clearer.

— The Newley introduced subsection on contributions help to better parse the framework in its entirety.

—Evaluation on newly added real-world datasets (JAAD, PIE) support the claims of the authors that the proposed framework adapts to real-world trajectory prediction.

— The elaboration on the description of evaluation metrics provide the reader with valuable guidance in interpreting the results.

—The newly added ablation studies help in assessing the effectiveness of the different parts of the framework.

—Addition of the description of the stepwise process and the improvements on Fig. 7 (previously Fig. 8) and the newly added Fig. 8 help in better understanding how the overall framework functions.

However, especially in Sec. 3, there is still a need for improvement. While the additions to Section 3 clearly improved its readability it is still quite dense and feels repetitive in some parts. I belief it could benefit from a clearer structure, e.g., by cross referencing relevant parts, and by a brief description of its structure in the beginning, to guide the reader through this rather lengthy section. Furthermore, as the descriptions in Sec 3.2 and the stepwise process in Sec 3.3 is highly related, authors may want to think about

the possibility to move Sec 3.3. to the beginning of Sec. 3.2 and cross reference the corresponding subsections in the descriptions of the steps.

It would also be good to have a clear indication and separation in Sec 3, of parts providing a more conceptual overview and those giving the technical details. For instance, Sec. 3.4 - 3.10 could be placed as sub subsections in a separate section on technical details.

Minor:

— *In section 3.3 the abbreviations (SIT, SAM, SFM) in the step heading, are a bit confusing, I belief they refer to the key methods used in each step, but this is not entirely clear.*

We thank you for your valuable feedback on Section 3. We agree that the section can be further improved in terms of structure and clarity. Below we outline the modifications we will make to address each of your suggestions.

1. Clearer structure and cross-referencing

We will add a short introductory paragraph at the beginning of Section 3 that outlines the overall organization: first a conceptual overview of the embodied cognition framework and its workflow (Sections 3.1–3.3), followed by detailed technical descriptions of each component (Sections 3.4–3.10). Throughout the section, we will insert cross-references (e.g., "as detailed in Section 3.4.1") to guide the reader between the high-level description and the corresponding technical subsections.

2. Reorganisation of Section 3.2 and Section 3.3

We agree that the stepwise integration workflow (currently Section 3.3) is closely related to the component descriptions in Section 3.2. To improve readability, we will move Section 3.3 ("Integration Workflow of the Embodied Cognition Framework") to the beginning of Section 3.2, right after the problem statement. This will provide an immediate high-level overview of how SAM, SIT, and SFM interact, and each step in the workflow will explicitly reference the corresponding technical subsections (e.g.,

"Step 1 (SAM) – see Section 3.4.1 for details"). This restructuring will make the flow more logical and reduce repetition.

3. Separation of conceptual overview and technical details

To clearly distinguish between conceptual explanations and mathematical/implementation details, we will group Sections 3.4–3.10 under a new subsection titled "3.4 Technical Implementation of Core Components", with each component described in a dedicated sub-subsection (e.g., 3.4.1 Social Force Model, 3.4.2 Scene-Aware Feature Extractors, …). This hierarchical structure will allow readers to easily navigate between the conceptual ideas and their concrete realizations.

4. Clarification of abbreviations in step headings

We understand that the abbreviations in the step headings of Section 3.3 (now moved to the beginning of Section 3.2) may be ambiguous. We will revise the headings to explicitly state the role of each component. For example, "Step 1: Sensory Filtering and Threat Prioritization (SAM)" already appears clear, but we will add a brief introductory sentence before listing the steps: "The framework operates in four sequential steps, each corresponding to one of the core embodied cognition modules:" followed by the enumerated steps. This will make it explicit that SAM, SIT, and SFM are the methods used in each step.

We believe these changes will significantly improve the readability and logical flow of Section 3, making it easier for readers to grasp both the conceptual framework and its technical implementation. Thank you again for your constructive suggestions.

Authors' Reply for NCOMMS-25-46440B

"Embodied Cognition-Driven Interpretable Trajectory Prediction of Autonomous Systems"

First, we sincerely thank the Editor-in-Chief, Associate Editor, and all reviewers for their constructive feedback, which has greatly improved our manuscript. We are pleased to provide a detailed point-by-point response below, outlining the specific revisions we have made in the updated manuscript.

Modification and Explanation for the Comments from Reviewer 3

Thank you for the detailed response and the added roll-out analysis. However, my main concern about the closed-loop evaluation remains. The new analysis is still open-loop and does not amount to true closed-loop evaluation with reactive agents. This is also reflected in the manuscript itself: while the limitations section acknowledges that the evaluation is not truly closed-loop, NCR is still described as a "closed-loop safety metric," Table 5 refers to a "closed-loop simulation protocol," and the paper claims to "close the perception-cognition-action loop." I therefore think these claims should be softened, and the added analysis should be framed as open-loop robustness or safety evaluation.

Regarding novelty, the response clarifies that the key contribution is the SIT-based PIG/VIG/DIG tri-graph design, and the added ablation supports the usefulness of these components. Still, this mostly strengthens the empirical support for the method rather than its claim to be fundamentally non-incremental. The overall framework still appears to be an integration of established components, with the main novelty lying in the interpretable SIT-based formulation. I therefore view the novelty as moderate.

Thank you for your valuable comments. Regarding the first concern about closed-loop evaluation, we fully agree that the current work has not achieved true closed-loop evaluation. The original expressions in the paper—such as "closed-loop safety metric", "closed-loop simulation protocol", and "closes the perception-cognition-action loop"—were indeed imprecise and potentially misleading. In the revised manuscript, we have corrected all related terminology: we redefine NCR as an "open-loop collision-aware safety metric", change the caption of Table 4 to "open-loop simulation protocol", and explicitly label the roll-out evaluation subsection as "open-loop roll-out analysis as a proxy for closed-loop robustness". In addition, we have added a clear statement in the Discussion/Limitations section, acknowledging that the current evaluation does not involve reactive agents and that true closed-loop

validation will be pursued in future work using interactive simulators such as CARLA or SMARTS. These revisions align the experimental description with the actual implementation and no longer overclaim closed-loop capabilities.

Regarding the second concern about novelty, we thank the reviewer for objectively pointing out that the novelty of this work is moderate. We have appropriately toned down overclaimed expressions such as “paradigm shift” in the Introduction and Discussion, and instead emphasize that the core contribution lies in **explicitly and computationally formalizing Social Impact Theory (SIT) for the first time into three trainable multi-relational graphs (PIG, VIG, DIG)**, and integrating them with a Scene Attention Mechanism (SAM) and a Social Force Model (SFM) into an interpretable, white-box trajectory prediction framework. Although individual components such as attention, graph neural networks, and social force models have been widely studied, their simultaneous integration under a unified cognitive-science framework (embodied cognition + SIT) and the use of interpretable graph weights to explain *why* a trajectory is predicted has not been shown in the existing literature. The ablation study further demonstrates that the joint use of the three graphs yields significant synergistic gains (28% ADE reduction), which goes beyond a simple stacking of existing components. We have added a new sentence in the Discussion: “While each of our building blocks has been explored individually, their integration under the explicit theoretical umbrella of embodied cognition and Social Impact Theory – with the tri-graph design enabling interpretable social reasoning – represents a non-trivial synthesis that advances the field toward more trustworthy autonomous systems.” We believe this positioning is both honest and reasonable, while still highlighting the practical value of our work in interpretability and efficiency.
